# National income inequality predicts cultural variation in mouth to mouth kissing

**DOI:** 10.1038/s41598-019-43267-7

**Published:** 2019-04-30

**Authors:** Christopher D. Watkins, Juan David Leongómez, Jeanne Bovet, Agnieszka Żelaźniewicz, Max Korbmacher, Marco Antônio Corrêa Varella, Ana Maria Fernandez, Danielle Wagstaff, Samuela Bolgan

**Affiliations:** 1 Division of Psychology, School of Social and Health Sciences, Abertay University, Dundee, DD11HG Scotland; 2 Facultad de Psicologia, Universidad El Bosque, Bogota, Colombia; 3 Institute for Advanced Study in Toulouse, Toulouse, France; 4 Department of Human Biology, University of Wroclaw, Wroclaw, Poland; 5 Department of Experimental Psychology, University of Sao Paulo, Sao Paulo, Brazil; 6 USACH, Escuela de Psicologia, University of Santiago, Santiago, Chile; 7 School of Health and Life Sciences, Federation University Australia, Churchill, Victoria, Australia

**Keywords:** Sexual selection, Human behaviour

## Abstract

Romantic mouth-to-mouth kissing is culturally widespread, although not a human universal, and may play a functional role in assessing partner health and maintaining long-term pair bonds. Use and appreciation of kissing may therefore vary according to whether the environment places a premium on good health and partner investment. Here, we test for cultural variation (13 countries from six continents) in these behaviours/attitudes according to national health (historical pathogen prevalence) and both absolute (GDP) and relative wealth (GINI). Our data reveal that kissing is valued more in established relationships than it is valued during courtship. Also, consistent with the pair bonding hypothesis of the function of romantic kissing, *relative* poverty (income inequality) predicts frequency of kissing across romantic relationships. When aggregated, the predicted relationship between income inequality and kissing frequency (*r* = 0.67, BCa 95% CI[0.32,0.89]) was over five times the size of the null correlations between income inequality and frequency of hugging/cuddling and sex. As social complexity requires monitoring resource competition among large groups and predicts kissing prevalence in remote societies, this gesture may be important in the maintenance of long-term pair bonds in specific environments.

## Introduction

Romantic love and passion are cultural universals^1,2^. Simultaneously, pair bonds within different cultures vary in their norms, rituals and forms of romantic expression^3^. In western samples, ‘ideal relationships’ are conceived on two dimensions of intimacy-loyalty and passion^4^, with intimacy related to relationship quality independent of couple sexuality^5^. Expressions of love and overt acts of affection enhance feelings of commitment^6^ and are related to stable marital bonds^7^. Moreover, sharing in novel and arousing activities enhances relationship quality^8^ and nonverbal expressions of love alter feeling states and facilitate the release of putative attachment hormones^9^. Collectively, many relationship behaviours and expressions of romantic attachment contribute to maintaining durable pair bonds, which are important to understand given that threats to pair bonds may affect health and wellbeing (see^10^ for discussion).

Although not a human universal, romantic kissing is observed in a wide variety of cultures^11^ and being perceived as a good kisser can enhance a person’s desirability as a partner (e.g., for short-term relationships^12^). Indeed, some naturalistic studies have documented kissing in courtship, where reciprocal affection is related to synchrony in body movements^13^. Cues obtained from close physical contact with a partner may facilitate mate assessment^14^, consistent with species that use gustatory and olfactory cues to regulate courtship rituals^15^ (see also^16^). As the prevalence of courtship rituals may point to their adaptive function, the mate assessment hypothesis^14,17^ proposes that kissing functions to assess putative cues to biological quality in a partner via close contact. In light of behavioural motivations to avoid pathogens (see^18^ for discussion), biases to over-perceive disease cues^19^, and given that the exchange of saliva may increase the likelihood of transmitting some infections (see^20^ for a recent review), romantic kissing may incur costs. As such, we do not engage in romantic kissing indiscriminately, unless the costs of kissing are traded off in favour of escalating courtship with a ‘high quality’ mate^14^. Consistent with the mate assessment hypothesis, kissing is more important, and is more likely to influence attraction, for the more ‘selective’ sex (women^21,22^), among more selective individuals (attractive individuals) and in contexts where the costs of choosing a less healthy mate are greater (i.e. in short-term sexual encounters^14^). The pair bonding hypothesis posits that, as an expression of love that strengthens romantic attachment, kissing plays a functional role in monitoring and maintaining long-term relationship quality^14^. Consistent with this hypothesis, kissing frequency and satisfaction with kissing are related to relationship quality while having a partner who is a good kisser predicts relationship quality at twice the size of the relationship between sexual satisfaction and relationship quality^17^. Collectively, kissing may be important both as a courtship custom and in maintaining stable long-term pair bonds.

Here, we extend Wlodarski & Dunbar’s^14,17^ two hypotheses, and test for cultural differences in the use and appreciation of kissing in romantic relationships. First, if kissing plays a role in assessments of ‘quality’, the benefits of assessing partner quality are likely to be greater in less-healthy environments (see, e.g.^23^, for a similar line of reasoning related to mate preferences). Here, we examine the mate assessment hypothesis and test whether people in less-healthy countries place greater importance on this form of courtship behaviour (i.e. to assess quality, as indexed via attitudes) but do so selectively (as indexed via frequency) in light of the greater potential risks of kissing within a high-pathogen environment. Thus, we predict that national health will be negatively related to the importance of kissing at the *initial* stages of a romantic relationship (Hypothesis #1) and, as a pathogen avoidance mechanism, will be negatively related to participant’s reported satisfaction with the *amount* of kissing in romantic relationships (i.e., a *weaker* desire for more frequent kissing in less-healthy countries, Hypothesis #2). Moreover, as the health costs of kissing in such environments will be lesser, we predict that national health will be positively related to the frequency of kissing within romantic relationships (Hypothesis #3). In other words, this would extend prior research by suggesting that the value attached to kissing as a mate assessment cue is greater in high pathogen ecologies (i.e., to accept or reject a partner) even if it is used sparingly in these environments.

Second, theoretical perspectives argue that monogamy and/or relationship investment are valued in harsh environments, such as those where resources are scarce in relative or absolute terms (see, e.g.^24,25^, for discussion), and the pair bonding hypothesis^14^ proposes that kissing plays an important role in how couples maintain and monitor the quality of a committed romantic relationship. Thus, we test the pair bonding hypothesis by examining whether individuals from countries of low absolute and relative wealth (i.e. high income inequality) place greater importance on kissing at the *established* (but not initial) phases of a romantic relationship (i.e. when investment is of greater concern; Hypothesis #4), report greater frequency of kissing in their relationships (to maintain a pair-bond in an economically harsh environment, Hypothesis #5) and lower satisfaction with the amount of kissing in their relationships (i.e., a stronger desire to signal investment through kissing in an economically harsh environment, Hypothesis #6).

When testing both hypotheses on the function of mouth-to-mouth kissing in relationships, we also test for national differences in two other forms of close/intimate contact (hugging and sexual intercourse) in order to examine whether our predictions are specific to, or are stronger or weaker for, mouth-to-mouth kissing than other forms of romantic expression. Evidence for specificity in our findings toward kissing would complement prior work, which demonstrates that kissing is a more substantial predictor of romantic relationship quality than other forms of closeness and/or intimacy such as sexual intercourse^17^, by suggesting that the ‘special’ role that kissing may play in the quality of long-term romantic relationships, and potentially relationship outcomes, varies according to the harshness of the environment. Evidence for stronger versus weaker relationships between ecological factors and different forms of intimacy could be interpreted in light of pathogen avoidance theories, given the greater potential costs to health of engaging in intercourse versus kissing.

Finally, we aim to replicate prior work by testing for an identical factor structure to the perceived components of a good kiss (biological/sensory component, arousal/contact and technique/execution^17^) among a broader international sample. We test whether factor scores on the biological component of a good kiss are negatively related to national health, which would demonstrate that biological factors, related to gustatory and olfactory cues, are a stronger component of a ‘good kiss’ in environments where exposure to pathogens is of greater concern (Hypothesis #7). We also run exploratory analyses on other anticipated factors (arousal/contact and technique/execution) and measures of national wealth to test whether factors related to synchrony, arousal and bonding during kissing are valued more in environments where an investing partner is particularly desirable.

## Individual Differences in Attitudes and Behaviours Related to Kissing and Intimacy (Replication of Prior Work)

A multilevel model (participant treated as a random effect) on the outcome variable *importance of kissing*, with the within subjects factor *relationship phase* (initial phase, established phase), the between subjects factor *participant sex* (male, female) and the covariates *self-rated attractiveness* and *participant age*, revealed a main effect of *relationship phase* (*F*(1,1906.0) = 18.84; *p* < 0.001) that was qualified by an interaction with *participant age* (*F*(1,1903.2) = 20.47; *p* < 0.001). The main effect of relationship phase reflected that participants, in general, thought kissing was more important in the established phases of a relationship (*M* = 84.40, 95% CI[83.53,85.27]) than it was in the initial phases of a relationship (*M* = 81.04, 95% CI[79.96,82.12], *t*(3669.8) = 4.76; *p* < 0.001, *d* = 0.15). To interpret the interaction between relationship phase and participant age, which was not predicted, we calculated a difference score where high scores indicate greater importance attached to kissing for the established versus initial phase of a relationship. Correlational tests against participant age revealed that younger people regarded kissing as more important in the established versus initial phase of a relationship compared to older respondents (*r*(2365) = −0.09; *p* < 0.001, BCa 95% CI [−0.13, −0.05]).

Main effects of *participant sex* (*F*(1,1905.3) = 5.05; *p* = 0.025), *participant age* (*F*(1,2331) = 61.94; *p* < 0.001, η_p_^2^ = 0.03) and *self-rated attractiveness* (*F*(1,2331) = 13.82; *p* < 0.001, η_p_^2^ = 0.006) were observed, with no other effects or interactions (both *F* < 2.49, both *p* > 0.11). Follow-up tests, collapsed across relationship phase, revealed that kissing was more important to older than younger people (*r* = 0.13; *p* < 0.001, 95% CI[0.10,0.16]), attractive participants (*r* = 0.05; *p* < 0.001, 95% CI[0.02,0.09]) and was more important to women (*M* = 83.09, 95% CI[82.30,83.89]) than to men (*M* = 81.70, 95% CI[80.29,83.10]; absolute *t*(1722.8) = 1.69; *p* = 0.09), although this difference was not significant in a two-tailed test.

**Figure 1:**
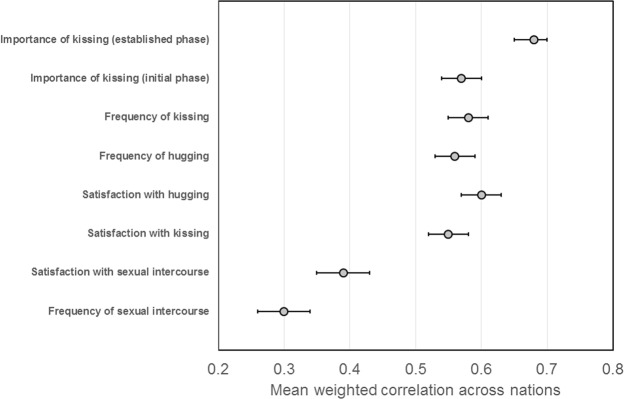
Mean weighted effect sizes (r) across nations for all items on our kissing questionnaire. Error bars show 95% Confidence Intervals.

Weighted effect sizes (see, e.g.^26^, for method) were generated for each country from one-sample *t* tests of responses to our scales against chance (i.e. 50). Large effects were observed across nations for all items, except for medium effects observed for frequency of/satisfaction with amount of sex (summarized in Fig. 1). National responses to five items did not differ from chance after correcting for multiple comparisons (104 comparisons, *p* < 4.8 × 10^−4^: Frequency of intercourse in Nigeria, India, and Australia; Importance of kissing at the initial phase of a relationship in Nigeria and sexual satisfaction in India).

### Cultural differences in attitudes and behaviours related to kissing and intimacy

All significant findings are presented in Table 1. When examining the importance of kissing, only *participant age* and *self-rated attractiveness* were positively related to the *importance of kissing at the initial phase of a romantic relationship* and also the *importance of kissing at the established phase of a romantic relationship*. No other relationships were significant in these two models (all absolute *t* < 2.21; all *p* > 0.03).

**Table 1:** Predictors of attitudes and behaviours related to kissing and romantic intimacy.

	Outcome variable	Significant predictors in full model	Est (b)	SE	*t*	*p*
Importance of kissing	**Initial phase of a relationship**					
		*Self-rated attractiveness*	2.06	0.45	4.61	<0.001^+^
		*Participant age*	0.33	0.05	6.84	<0.001
	**Established phase of a relationship**					
		*Self-rated attractiveness*	1.28	0.37	3.45	<0.001
		*Participant age*	0.14	0.04	3.34	<0.001
Frequency	**Kissing**					
		*GINI*	0.37	0.11	3.47	<0.01^+^
		*Self-rated attractiveness*	1.78	0.39	4.53	<0.001
		*Relationship status*	−4.55	1.10	−4.15	<0.001
	**Hugging/cuddling (without kissing)**					
		*Relationship status*	−5.85	1.22	−4.80	<0.001
	**Sex**					
		*Self-rated attractiveness*	2.92	0.47	6.21	<0.001
Satisfaction (with amount)	**Kissing**					
		*Participant age*	−0.17	0.05	−3.58	<0.001
		*Self-rated attractiveness*	1.51	0.43	3.52	<0.001
		*Relationship status*	−4.05	1.21	−3.34	<0.001
	**Hugging/cuddling (without kissing)**					
		*Participant age*	−0.26	0.05	−5.92	<0.001
		*Self-rated attractiveness*	1.28	0.41	3.12	= 0.002
		*Relationship status*	−7.50	1.14	−6.57	<0.001
	**Sex**					
		*Participant sex*	5.53	1.40	3.96	<0.001
LMM on PCA results	**Technique component of a ‘good kiss’**					
		*Participant age*	0.006	0.002	2.70	= 0.007
	**Sensory component of a ‘good kiss’**					
		*Participant age*	0.02	0.002	7.43	<0.001
		*Participant sex*	0.29	0.05	5.64	<0.001

*α* = 0.007 After correcting for multiple comparisons in full model [7 comparisons, *α* = 0.008 for PCA models]. ^+^Denotes support for pre-registered hypothesis or replication of prior effect.

When examining the frequency of three romantic behaviours, consistent with Hypothesis #5, *GINI coefficient* was positively related to the *frequency of kissing in romantic relationships*. *Self-rated attractiveness* and *relationship status* were also related to this outcome variable. Only *relationship status* predicted *frequency of hugging/cuddling in romantic relationships* and only *self-rated attractiveness* predicted *frequency of sexual intercourse in romantic relationships*. Although *historical pathogen prevalence* (*Est(b)* = −8.37, *SE* = 2.33, *t*(7.8) = −3.60; *p* = 0.0072) and *GDP* (*Est(b)* = −0.3, *SE* = 0.11, *t*(10.37) = −2.69; *p* = 0.02) were related to hugging frequency in the predicted direction, they were not significant after correcting for multiple comparisons. No other relationships were significant in these three models (all absolute *t* < 2.70; all *p* > 0.02).

**Figure 2:**
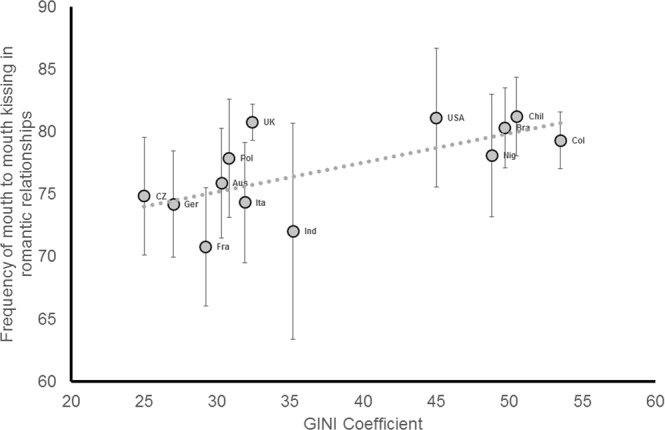
Income inequality predicts national differences in frequency of mouth to mouth kissing in romantic relationships (*r* = 0.67, BCa 95% CI[0.32,0.89]).

Correlational tests were run to visualize the significant relationship in support of Hypothesis #5 (aggregated across country). As predicted, GINI coefficient was correlated with kissing frequency (*r*(13) = 0.67; *p* = 0.012, BCa 95% CI[0.32,0.89], see Fig. 2). By way of comparison, there was no correlation between GINI and frequency of hugging (*r*(13) = 0.12; *p* = 0.69) or sexual intercourse (*r*(13) = 0.13; *p* = 0.68), nor was there a relationship between absolute wealth (GDP) and kissing frequency (*r*(13) = −0.06; *p* = 0.85). In response to comments from Reviewers, we also reanalysed this set of three tests on frequency of romantic behaviours, treating GINI as a categorical variable (*N* = 1204 participants from countries of lower inequality and *N* = 660 participants from countries of greater inequality). These two groupings reflected participants within the top 26% of high inequality nations and within the top 40% of low inequality nations as ranked by the CIA world fact book. A univariate ANCOVA on the outcome variable *frequency of kissing in romantic relationships*, with the between subjects factor *GINI grouping* (less-equal nation, more-equal nation) and the covariates *participant age*, *participant sex*, *relationship status* and *self-rated attractiveness* revealed a significant effect of *GINI grouping* (*F*(1,1869) = 6.19; *p* = 0.013, η_p_^2^ = 0.003) in the same direction as our mixed models and aggregated analyses. No equivalent effect of GINI grouping was observed when running the ANCOVA on the outcome variables *frequency of hugging/cuddling in romantic relationships* (*F*(1,1858) = 3.64; *p* = 0.057, η_p_^2^ = 0.002) or *frequency of sexual intercourse in romantic relationships* (*F*(1,1830) = 0.29; *p* = 0.59).

When examining satisfaction with the amount of these three behaviours across participants’ romantic relationships, *GINI coefficient* was positively related to *satisfaction with the amount of kissing in romantic relationships* (i.e. in the opposite direction to our prediction for Hypothesis #6). Satisfaction with the amount of kissing and also the amount of hugging/cuddling in relationships were predicted by *participant age*, *self-rated attractiveness* and *relationship status*. Only *participant sex* predicted *satisfaction with the amount of sexual intercourse in romantic relationships* after correcting for multiple comparisons, which reflected less satisfaction among men (*M* = 67.69, 95% CI[65.14,70.24]) than among women (*M* = 73.17, 95% CI[71.83,74.51], *t*(780.54) = 3.74; *p* < 0.001, *d* = 0.21). No other relationships were significant in these three models (all absolute *t* < 2.78; all *p* > 0.013). Finally, when examining predictors of the importance of the two components of a ‘good kiss’ generated from our Principal Components Analysis, *participant age* was positively related to the importance of both the ‘technique, contact and arousal’ component and the ‘sensory component’ of a good kiss. *Participant sex* was related to the importance of the *sensory component of a good kiss*, which reflected greater importance of this component among women (*M* = 0.09, 95% CI[0.04,0.14]) than among men (*M* = −0.15, 95% CI[−0.25, −0.05], absolute *t*(770.49) = 4.28; *p* < 0.001, *d* = 0.24). No other relationships were significant in these two models (all absolute *t* < 2.20; all *p* > 0.04).

## Discussion

Our research across thirteen nations from six continents reveals that kissing is more important at later established phases of a romantic relationship than during courtship, with this effect stronger for younger participants. These data support the pair-bonding hypothesis^14^. Replicating prior work^14^, albeit with small effects, women and attractive individuals viewed kissing as more important in relationships than men and less attractive individuals, and attractive people reported more kissing and sex in relationships and greater satisfaction with the amount of kissing and hugging in their relationships. Older participants placed greater importance on kissing in romantic relationships and on the associated components of a ‘good kiss’ than younger individuals. Moreover, they were less satisfied with the amount of kissing and hugging in their relationships. As many of our items gauged trait-level opinions (i.e. across the participant’s relationships) these findings may merit further research into generational differences in romantic intimacy.

In further support of the pair-bonding hypothesis, income inequality was positively related to kissing frequency (according to Hypothesis #5), suggesting that people in areas of greater inequality kiss their partner more often. No relationships were observed between absolute wealth and kissing behaviours or attitudes. To illustrate that the effect of income inequality was specific to *kissing* frequency, aggregated analyses revealed that the correlation in support of Hypothesis #5 (more kissing in nations of high income inequality than nations of low income inequality) was over five times the size of null correlations between GINI and frequency of hugging and sex. This specific relationship between GINI and kissing frequency was also observed when treating income inequality as a categorical variable. Collectively, our data suggest a robust relationship between *relative* poverty and greater kissing frequency.

In contrast to recent work^27^, support for the mate assessment hypothesis in predicting cultural differences in kissing and intimacy was limited. We observed no relationships between historical pathogen prevalence and kissing, hugging or sex after correcting for multiple comparisons within a model, although (before alpha correction) pathogen prevalence and absolute poverty predicted hugging frequency in the expected direction (i.e. greater hugging in healthier and poorer nations). Consistent with the mate assessment hypothesis, and replicating prior work, principal components analysis of the components of a ‘good kiss’ generated one of two components representing *sensory factors*, which were more important to women than to men. In addition, ‘pleasantness of breath’ was the most important aspect of a good kiss more generally, consistent with^17^. Although preliminary, as the effect was not robust, the sensory component of a ‘good kiss’ was more important to participants in less-equal countries (i.e. related to GINI before controlling for demographic variables), and the technique/arousal component was more important to participants in less-healthy countries, before controlling for wealth and demographic variables (see Supplemental Materials). This pattern of preliminary findings was in contrast to that predicted for Hypothesis #7, but is consistent with prior work where sexual arousal lowers disgust sensitivity (see^28^ for discussion), which presumably would be important for intimacy in high-pathogen ecologies, and may suggest that the experience of a kiss is embodied within one’s environment. Further direct tests of interactions between chemosensory cues, relationship context and environment on kissing behaviours and attitudes may prove fruitful. Moreover, in light of recent work on pathogen prevalence and cultural norms that foster close contact, evidenced by the presence or absence of kissing in a culture^27^, our findings develop work by suggesting that health factors may not be a powerful motivator of cultural *variation* in the strength of behaviours and attitudes related to kissing. Work from different approaches (experimental and regional differences and interactions between individual-level and country-level factors) is still warranted to examine the mate assessment and pair bonding hypotheses of the function of romantic kissing.

Our research has potential limitations. For aggregated analyses, caution should be drawn in making strong inferences from data aggregated across 13 nations, although findings converged when treating inequality as a dichotomous variable, which suggest that these data may not be an artefact of comparing the ‘west versus the rest’^29^. In light of previous discussion^29^, it is important to note that we do not claim causal effects of the environment on individual romantic behaviour, nor do our data speak to variation in kissing in light of one’s own health or wealth status. Of note, our published questionnaire items (as outcome variables) were not suited to include additional individual-level variables within our models, as observed relationships between *current* health/wealth and attitudes/use of kissing across different relationships would be difficult to interpret. However, our research also meets some important guidelines for robustness in cross-cultural work^30^. For example, preliminary models that demonstrate relationships between health, wealth and hugging are consistent with data from the anthropological record (i.e., remote cultures within nations) which demonstrates negative relationships between pathogen prevalence and the level of physical contact made during greetings^27^. This increases confidence that our findings are not mere artefacts of testing a ‘WEIRD’ sample^31^ (see also^30^) with access to the internet. Finally, although the ‘reference group effect’ (e.g.^32^) is important in cross-cultural research, two factors increase confidence in our findings. First, we replicate findings from an ethnically homogenous sample^14,17^. Second, relationships between GINI and kissing but no other romantic behaviours may suggest that our findings are not artefacts of group-level differences in how participants use different ends of a response scale. Of course, further work examining cultural differences in latent factors derived from surveys of similar behaviours is of utility for relationship and health sciences.

Our findings raise implications about romantic passion in the form of a kiss, within human pair-bonds. Although kissing, in contrast to romantic passion, is not a human universal^11^ (see also^33^) data from remote societies suggests that social complexity, indexed via stratification, predicts the likelihood of observing romantic kissing in that culture^11^ (same dataset as used in^27^). Social complexity and increased group competition for scarce resources was thought to be a key driver in the evolution of human social intelligence, selecting for those who could monitor relations between group members as groups increased in size^34^. Evidence that relative poverty predicts kissing frequency raises the possibility that this gesture evolved in humans to maintain pair bonds in light of the constraints of that environment. Behaviours related to kissing are a stronger predictor of relationship quality than other forms of intimacy^17^. Thus, kissing may play an important role in maintaining long-term pair bonds (i.e., communal relationships^35^) in ecologies where investment and monogamy are valued.

In sum, across thirteen countries, we show both individual (age, own-attractiveness, sex) and environmental differences (income inequality) in attitudes and/or behaviours related to kissing in romantic relationships and the perceived factors involved in a ‘good kiss’. Individuals kiss their partner more in countries where resource competition is likely to be more intense, which may play an important role in maintaining long-term stable pair bonds in certain types of harsh environment.

## Methods

### Participants

Three-thousand one hundred nine participants (*M*_age_ = 31.90 years, *SD* = 11.60 years) were recruited to an online study. Data collection ended after collating data from 13 countries (6 continents), with countries included in cross-national comparisons if we obtained data from at least 50 respondents (who reside in the same country as their birth) and responses to our kissing questionnaire from at least 30 women. Participants were recruited from campuses and the wider community, research participant pools, word of mouth, twitter, academic groups on social media and a press release from the lead author’s communications department. This press release informed readers that we were conducting a global study into how people across cultures express themselves in romantic relationships, but did not mention national health or national wealth. Given difficulty in recruiting an African sample, a sample of Nigerians were recruited via the buy responses function on surveymonkey.com. No participants were reimbursed for their time. Duplicate responses from the same device were not permitted by the survey platform.

All procedures for testing and recruitment were approved via the lead author’s Ethics Committee (School of Social and Health Sciences, Abertay University) and run in accordance with the ethical principles and guidelines of the British Psychological Society. Hypotheses, methods and analyses were pre-registered (https://osf.io/pbqwm/). All aspects of the pre-registration report are identical to the current manuscript, except for aspects of the analytical strategy following discussion between the first, second and third authors in response to valuable comments from the Reviewers about the suitability of our data for analyses via LMEM. Participants provided informed consent after reading an information sheet describing the contents of the survey. We excluded participants who i) reported being less than 18 years old, ii) did not report their sex as male or female, or, for cross-cultural analyses, iii) if their IP address did not match their reported country of residence (following^36^). The final sample size was 2988 participants (794 males, *M*_age_ = 32.01 years, *SD* = 11.56 years, 72% in a long-term romantic relationship, 89% reported their orientation as heterosexual), 2379 of whom were eligible for analyses comparing nations (643 males, *M*_age_ = 32.34 years, *SD* = 11.85 years, 71% in a long-term romantic relationship, 89% reported their orientation as heterosexual, see Table 2).

**Table 2:** Descriptive statistics and r values for participants eligible for cross-cultural analyses.

Country	Language	*N*	Males	*M*_age_	r values							
					Importance		Frequency			Satisfaction (with amount)		
					Initial phase	Established phase	Kissing	Hugging	Sex	Kissing	Hugging	Sex
Australia	English	120	39	30	0.44	0.65	0.51	0.51	0.14	0.46	0.60	0.22
Brazil	Portuguese	208	69	29	0.55	0.67	0.58	0.48	0.29	0.57	0.65	0.34
Chile	Spanish	172	37	31	0.56	0.69	0.65	0.69	0.31	0.59	0.57	0.42
Colombia	Spanish	284	88	26	0.53	0.64	0.64	0.65	0.38	0.64	0.62	0.49
Czech Republic	English	85	19	27	0.54	0.59	0.53	0.58	0.27	0.50	0.54	0.37
France	French	100	25	32	0.64	0.46	0.42	0.49	0.36	0.45	0.56	0.46
Germany	German	90	12	28	0.68	0.66	0.54	0.69	0.26	0.67	0.66	0.44
India	English	62	7	29	0.42	0.56	0.36	0.41	0.05	0.47	0.39	0.22
Italy	Italian	65	8	30	0.67	0.83	0.56	0.30	0.44	0.59	0.52	0.45
Nigeria	English	99	37	32	0.10	0.48	0.50	0.49	0.04	0.62	0.55	0.32
Poland	Polish	106	27	28	0.64	0.71	0.54	0.77	0.37	0.55	0.60	0.38
United Kingdom	English	916	256	38	0.63	0.73	0.61	0.53	0.32	0.51	0.60	0.38
United States	English	73	19	33	0.43	0.67	0.59	0.43	0.25	0.65	0.70	0.42

R values calculated as in Fig. 1. For each questionnaire item below, responses ranged from 41–726.

### Measures

Participants first provided demographic information and proxies for ‘mate quality’ (sex, age, sexual orientation, country of residence, country of birth, relationship status, relationship length, ethnicity, self-rated attractiveness, self-rated masculinity, self-rated health, rated attractiveness/masculinity/health of current romantic partner) and then completed three questionnaires in a randomized order. Two of these questionnaires were run as part of a separate project. Attractiveness, masculinity and health of self/partner were measured on a 1 (much less than average) to 7 (much more than average) scale.

Participants completed a questionnaire about their attitudes toward mouth-to-mouth kissing (adapted from^14,17^). Participants were informed that we were interested in their views on various behaviours that a person may engage in with a romantic partner, whom we defined as a person with whom you could be romantically involved with or without the involvement of sex. Kissing was defined as kissing on the lips or open-mouth (i.e. ‘French’ kissing). Participants were asked how important they thought kissing was i) at the very initial stages of a romantic relationship and ii) during the established phases of a committed, long-term relationship on a 0 (not at all important) to 100 (extremely important) scale, with their choice on the scale visible. Participants were asked, in general, when they are in a romantic relationship i) how often they kiss their partner, ii) just hug or cuddle their partner (a single hug or cuddle without kissing involved), iii) have sexual intercourse with their partner, on a 0 (not at all) to 100 (very often) scale. Participants indicated their satisfaction with the amount of kissing/hugging-cuddling/sexual intercourse (in general, when they are in a romantic relationship) on a 0 (not at all satisfied) to 100 (very satisfied) scale.

Finally (*sensu*^17^), participants indicated the importance of seven factors when deciding whether someone is a ‘good kisser’ on a 0 (not at all important) to 100 (extremely important) scale: i) *How pleasant their breath is* (*M* = 86.74, *SD* = 19.18), ii) *The scent of their body* (*M* = 83.05, *SD* = 19.90), iii) *The taste of their lips/skin* (*M* = 76.25, *SD* = 24.49), iv) *How ‘wet’ the kiss is* (*M* = 65.11, *SD* = 27.27), v) *How much touching/physical-contact/caressing is involved* (*M* = 76.62, *SD* = 21.91), vi) *How physically aroused it makes you* (*M* = 74.07, *SD* = 25.00), vii) *Whether their kissing style is the same as yours* (*M* = 65.91, *SD* = 27.86). After completing all questionnaires, participants were debriefed and could exit the survey. Native speakers based at a university translated foreign language versions of the study (French, Spanish, Portuguese, Italian, German, and Polish).

We measured national differences in parasite stress using the historical prevalence of pathogens within regions (9-item measure^37^) which is strongly correlated with other estimates of parasite stress (see^38^). Higher scores indicate greater historical prevalence of pathogens. National differences in absolute wealth and income inequality (e.g.^39,40^) were measured via national GDP per capita and the GINI Index (inequality in the distribution of family income) obtained from the CIA World Factbook in March 2018 (https://www.cia.gov/library/publications/the-world-factbook/). High scores indicate greater absolute wealth and greater income inequality respectively. Of the countries sampled at the time of analysis, GINI coefficients ranged from 10^th^ in the world to 150^th^ in the world (GDP range from 3^rd^ to 50^th^ in the world).

### Analytical strategy

#### Models

First, general linear models were run across the entire sample, to replicate prior work on differences in attitudes toward kissing at different relationship phases, as a function of the participant’s sex and self-rated attractiveness^14^. Cultural differences were examined on R (version 3.5.2) using Linear Mixed-Effects Models and Principal Components Analysis (with varimax rotation). All linear mixed-effects models were built in the same manner to test each hypothesis, using restricted maximum likelihood criterion and Satterthwaite’s method for t tests, nested within the higher-level variable of country (random intercept). A first model includes the predictor Historical Pathogen Prevalence, followed by a second model which includes GINI and GDP (GDP scaled in thousands) as additional predictor variables. A final (third) model includes these three ecological variables and control variables. In these models we control for participant sex and self-rated attractiveness (in light of^14^) by including it in our model, and, in addition, control for relationship status and participant age in light of demographic differences between our sampled nations (following^38^). Self-rated attractiveness was not included in a third model to test Hypothesis # 7, given no a priori reason to do so. With this exception, all full models thus took the form of: *Outcome variable ~ Historical Pathogen Prevalence* + *GINI* + *GDP* + *Participant Sex* + *Participant Age* + *Self-rated Attractiveness* + *Relationship Status* + *(1*/*Country)*. Significant findings from these full models are reported in the main text after correcting for multiple comparisons. Relationships confirming hypotheses are illustrated via scatterplots with outcome variables aggregated at the national level (Fig. 2). Multicollinearity was not a problem according to established rules of thumb (see^41^) for all predictors in cross-cultural analyses. No Variance Inflation Factor statistics were greater than 10, the average VIF was not substantially greater than 1 and no tolerance statistics were below 0.2 (Average VIF = 2.11, Largest VIF = 4.49, All tolerance statistics >0.22). Tables displaying results from first and second models, and a full r markdown file with all models, are included as Supplemental Materials.

*PCA on the components of a ‘good kiss’*. Principal components analysis on answers from the entire eligible sample to the seven items contributing to a “good kiss” generated two components. A component labelled ‘*technique, contact and arousal*’ and a second component, labelled ‘*sensory factors*’ consistent with Wlodarski & Dunbar^17^ (variance explained = 56.80%). Sampling adequacy was verified by the Kaiser-Meyer-Olkin technique (KMO = 0.75) with Bartlett’s test of sphericity indicating significantly large correlations (χ^2^(21) = 3204.53, *p* < 0.001). Rotated coefficients for the sensory component (‘Pleasantness of breath’ = 0.82, ‘Scent of body’ = 0.84, ‘Taste of lips/skin’ = 0.73) and the technique, contact and arousal component (‘Wetness of kiss’ = 0.59, ‘Touching, physical contact and caressing’ = 0.75, ‘Physical arousal’ = 0.78, ‘Similar technique of partner’ = 0.60) all exceeded 0.5 (all rotated coefficients on opposite component <0.27).

## Supplementary information

Supplemental material

Markdown file for analyses

Dataset 1

Dataset 2

Dataset 3

## Data Availability

Data will be uploaded onto the Open Science Framework upon publication with an accompanying codebook (https://osf.io/pbqwm/).

## References

[1] Jankowiak, W. R. *Romantic Passion: A Universal Experience*? New York: Columbia University Press (1995).

[2] Jankowiak, W. R. *Intimacies: Love and Sex across Cultures*. New York: Columbia University Press (2008).

[3] Hatfield, E. & Rapson, R. L. *Love and Sex: Cross-cultural perspectives*. Lanham: University Press of America (2005).

[4] Fletcher GJO, Simpson JA, Thomas G, Giles L (1999). Ideals in intimate relationships. J Person Soc Psychol..

[5] Hassebrauck M, Fehr B (2002). Dimensions of relationship quality. Pers Relatsh..

[6] Marston PJ, Hecht ML, Manke ML, Mcdaniel S, Reeder H (1998). The subjective experience of intimacy, passion, and commitment in heterosexual loving relationships. Pers Relatsh..

[7] Huston TL, Caughlin JP, Houts RM, Smith SE, George LJ (2001). The connubial crucible: Newlywed years as predictors of marital delight, distress, and divorce. J Person Soc Psychol..

[8] Aron A, Norman CC, Aron EN, McKenna C (2000). Couples’ shared participation in novel and arousing activities and experienced relationship quality. J Person Soc Psychol..

[9] Gonzaga GC, Turner RA, Keltner D, Campos B, Altemus M (2006). Romantic love and sexual desire in close relationships. Emotion..

[10] Cacioppo JT, Cacioppo S, Capitanio JP, Cole SW (2015). The neuroendocrinology of social isolation. Annu Rev Psychol..

[11] Jankowiak WR, Volsche SL, Garcia JR (2015). Is the Romantic-Sexual Kiss a Near Human Universal?. Am Anthropol..

[12] Wlodarski R, Dunbar RIM (2014). What’s in a Kiss? The Effect of Romantic Kissing on Mate Desirability. Evol Psychol..

[13] Brak-Lamy G (2015). Heterosexual Seduction in the Urban Night Context: Behaviors and Meanings. J Sex Res..

[14] Wlodarski R, Dunbar RIM (2013). Examining the possible functions of kissing in romantic relationships. Arch Sex Behav..

[15] Clowney EJ, Iguchi S, Bussell JJ, Scheer E, Ruta V (2015). Multimodal Chemosensory Circuits Controlling Male Courtship in Drosophila. Neuron..

[16] Thistle R, Cameron P, Ghorayshi A, Dennison L, Scott K (2012). Contact Chemoreceptors Mediate Male-Male Repulsion and Male-Female Attraction during Drosophila Courtship. Cell..

[17] Wlodarski R, Dunbar RI (2013). Menstrual cycle effects on attitudes toward romantic kissing. Hum Nat..

[18] Tybur JM, Lieberman D (2016). Human pathogen avoidance adaptations. Curr Opin Psychol..

[19] Miller SL, Maner JK (2012). Overperceiving disease cues: The basic cognition of the behavioral immune system. J Person Soc Psychol..

[20] Posse, J. L., Dios, P. D. & Scully, C. Infection Transmission by Saliva and the Paradoxical Protective Role of Saliva. *Saliva Protection and Transmissible Diseases*, 1–18 (2017).

[21] Todd PM, Penke L, Fasolo B, Lenton AP (2007). Different cognitive processes underlie human mate choices and mate preferences. Proc Natl Acad Sci USA.

[22] Trivers, R. L. Parental investment and sexual selection. In Campbell, B. (Ed.), Sexual selection and the descent of man, 1871–1971 (pp. 136–179). London: Heinemann (1972).

[23] Gangestad SW, Haselton MG, Buss DM (2006). Toward an integrative understanding of evoked and transmitted culture: the importance of specialized psychological design. Psychol Inq..

[24] Gangestad SW, Simpson JA (2000). The evolution of human mating: Trade-offs and strategic pluralism. Behav Brain Sci..

[25] Little AC, Jones BC, DeBruine LM (2011). Facial attractiveness: Evolutionary based research. Philos Trans R Soc Lond B Biol Sci..

[26] Pisanski K (2014). Vocal indicators of body size in men and women: a meta-analysis. Anim Behav.

[27] Murray DR, Fessler DMT, Kerry N, White C, Marin M (2017). The kiss of death: Three tests of the relationship between disease threat and ritualized physical contact within traditional cultures. Evol Hum Behav.

[28] Thornhill, R. & Fincher, C. L. *The Parasite-Stress Theory of Values and Sociality*. Springer: London (2014).

[29] Pollet TV, Tybur JM, Frankenhuis WE, Rickard IJ (2014). What can cross-cultural correlations teach us about human nature?. Hum Nat.

[30] Hruschka DJ, Hackman J (2014). When are cross-group differences a product of a human behavioral immune system?. EBS.

[31] Henrich J, Heine SJ, Norenzayan A (2010). The weirdest people in the world?. Behav Brain Sci.

[32] Milfont TL, Fischer R (2010). Testing measurement invariance across groups: Applications in cross-cultural research. Int J Psychol Res.

[33] Sorokowski P (2017). Love influences reproductive success in humans. Front Psychol..

[34] Flinn MV, Geary DC, Ward CV (2005). Ecological dominance, social competition, and coalitionary arms races: Why humans evolved extraordinary intelligence. Evol Hum Behav.

[35] Fiske AP (1992). The four elementary forms of sociality: Framework for a unified theory of social relations. Psychol Rev.

[36] DeBruine LM, Jones BC, Crawford JR, Welling LLM, Little AC (2010). The health of a nation predicts their mate preferences: cross-cultural variation in women’s preferences for masculinized male faces. Proc Roy Soc Lond B Biol Sci..

[37] Murray DR, Schaller M (2010). Historical prevalence of infectious disease within 230 geopolitical regions: A tool for investigating origins of culture. J Cross Cult Psychol..

[38] Tybur JM (2016). Parasite stress and pathogen avoidance relate to distinct dimensions of political ideology across 30 nations. Proc Natl Acad Sci USA.

[39] Brooks R (2011). National income inequality predicts women’s preferences for masculinized faces better than health does. Proc Roy Soc Lond B Biol Sci..

[40] Daly M, Wilson M, Vasdev S (2001). Income inequality and homicide rates in Canada and the United States. Can J Criminol Crim Justice..

[41] Field, A. F. *Discovering statistics using IBM SPSS Statistics*. Sage: London (2018).

