# Supplementary material for: National income inequality predicts cultural variation in mouth to mouth kissing
Source: Sci Rep. 2019 Apr 30;9:6698. doi: 10.1038/s41598-019-43267-7 (PMC6491799; doi:10.1038/s41598-019-43267-7)
Supplement: Supplementary file 1 — Supplemental material [file 41598_2019_43267_MOESM1_ESM.pdf]

National income inequality predicts cultural variation in mouth to mouth kissing

CHRISTOPHER D WATKINS[1*], JUAN DAVID LEONGÓMEZ[2], JEANNE BOVET[3], AGNIESZKA ŻELAŹNIEWICZ[4], MAX KORBMACHER[1], MARCO ANTÔNIO CORRÊA VARELLA[5], ANA MARIA FERNANDEZ[6], DANIELLE WAGSTAFF[7] & SAMUELA BOLGAN[1]

[1]Division of Psychology, School of Social and Health Sciences, Abertay University, Dundee, Scotland DD11HG

[2]Facultad de Psicologia, Universidad El Bosque, Bogota, Colombia

[3]Institute for Advanced Study in Toulouse, Toulouse, France

[4]Department of Human Biology, University of Wroclaw, Wroclaw, Poland

[5]Department of Experimental Psychology, University of Sao Paulo, Sao Paulo, Brazil

[6]USACH, Escuela de Psicologia, University of Santiago, Chile

[7]School of Health and Life Sciences, Federation University Australia, Churchill, Victoria, Australia

***Corresponding author:**

Dr Christopher Watkins

Division of Psychology, School of Social and Health Sciences,

Abertay University, Dundee, Scotland DD11HG

Tel: (44)1382 308646

Supplemental material for Watkins et al. National income inequality predicts cultural variation in mouth to mouth kissing

Table S1. Significant results from first (pathogen prevalence) and second models (national health and wealth). See markdown file for full results.

	Outcome variable	Significant predictors	Est (b)	SE	t	p
	**First model (Historical pathogen prevalence only)**					
LMM on PCA results	**Technique component of a good kiss**					
		HPP_9	0.22	0.05	4.39	<.01
	**Second model (Historical Pathogen Prevalence, GINI and GDP)**					
Frequency	**Kissing**					
		GINI	0.32	0.10	3.22	<.01
	**Hugging/cuddling (without kissing)**					
		HPP_9	-8.27	2.22	-3.72	<.01
		GDP	-0.32	0.11	-2.93	=.01
Satisfaction (with amount)	**Sex**					
		GINI	0.27	0.11	2.38	=.04
LMM on PCA results	**Sensory component of a 'good kiss'**					
		GINI	0.01	0.005	2.59	=.03

Supplemental material for Watkins et al. National income inequality predicts cultural variation in mouth to mouth kissing

Codebook for data files (all sample & cross-cultural comparisons)
Watkins et al.
[see manuscript for exact instructions given to participants on questionnaire items]

Data File #1 - Kissing_submitted data (main data file from which all analyses were derived)

ID
Ex_Sample - If=1, indicates that participant should be excluded from initial analyses
Ex_Cross - If=1, indicates that participant should be excluded from cross-national comparisons
Consent
Sex
SexPref - Sexual orientation
Age (in years)
Country_Res - country where they live
Country_Born

HPP_9 - Historical Pathogen prevalence (Schaller & Murray, 2010)
GDP - Gross domestic product (CIA World Factbook)
GINI - Income inequality (high scores = high income inequality, CIA World Factbook)
SexRatio(2017est) - Total national sex ratio estimated for 2017 (CIA World Factbook)
SexRatio(15-54) - National sex ratio averaged across two age cohorts (CIA World Factbook)
UNRegion - UN regions dummy coded (1=Africa, 2=Asia-Pacific, 3=E Europe, 4=Latin America and Caribbean, 5=W Europe & Ohters)

RelStatus - Whether the participant considered themselves in a long term romantic relationship (1=Yes, 2=No)
RelLength (in years - calculated from RelY+(RelM/12))

Ethnicity
SRA - Self-rated attractiveness (1-7 scale, high scores = high attractiveness comapred to average)
SRM - Self-rated masculinity (1-7 scale, 7 = high masculinity)
SRH - Self-rated health (1-7 scale, high scores = high health)
SRA_Partner - Rated attractiveness of partner (1-7 scale)
SRM_Partner - Rated masculinity of partner (1-7 scale)
SRH_Partner - Rated health of partner (1-7 scale)

Kiss_Imp_Initial - Importance of kissing at the initial phases of a relationship
Kiss_Imp_Est - Importance of kissing at the established phases of a relationship
Kiss_Freq - Frequency of kissing, in general, within romantic relationships
Hug_Freq - Frequency of hugging/cuddling (without kissing), in general, within romantic relationships
Sex_Freq - Frequency of sex, in general, within romantic relationships

Kiss_Satis - Satisfaction with amount of kissing, in general, within romantic relationships
Hug_Satis - Satisfaction with amount of hugging/cuddling, in general, within romantic relationships
Sex_Satis - Satisfaction with amount of sexual intercourse, in general, within romantic relationships

Constituents of a 'good kiss'
GK_Breath - Pleasantness of breath
GK_Scent - Scent of their body
GK_Taste - Taste of lips/skin
GK_Wet - Wetness of kiss
GK_Contact - Involvement of touching/physical contact/caressing
GK_Arous - Physical arousal
GK_SynchStyle - Same kissing style (partner)

Data File #2 - Aggregated_Correlation_tests
Follow up tests where mean scores were aggregated across a given nation

Data File #3 - Calculations Meta Analysis_Fig 1
Calculations that lead to Figure 1. We followed the calculations/method reported in Pisanski et al. 2014 Animal Behaviour who, in turn, follow Hedges & Olkin, 1985 and Lipsey & Wilson, 2001.
Calculations are based on effect size r (one sample t tests comparing the mean scores on each item against chance on the measurement scale (i.e. 50), averaged across nations and corrected for multiple comparisons).
