# Supplementary material for: National income inequality predicts cultural variation in mouth to mouth kissing
Source: Sci Rep. 2019 Apr 30;9:6698. doi: 10.1038/s41598-019-43267-7 (PMC6491799; doi:10.1038/s41598-019-43267-7)
Supplement: Supplementary file 2 — Markdown file for analyses [file 41598_2019_43267_MOESM2_ESM.pdf]

Code and analyses for *National income inequality predicts cultural variation in mouth to mouth kissing*

Jeanne Bovet, Juan David Leongómez & Christopher D. Watkins

Contents

Preliminaries 1
- Import packages 1
- Loading data 1
- Factors 2
- Rescale variable GDP 2
- Excluding participants 2

Individual differences in attitudes and behaviours related to kissing and intimacy 2
- Mixed model 2
- Follow-up tests 2

Multilevel models 4
- Models Importance of kissing at initial phase 4
- Models Importance of kissing at established phase 7
- Models frequency of kissing 11
- Models frequency of hugging 14
- Models sex frequency 17
- Models kissing satisfaction 20
- Models Huging satisfaction 24
- Models Sex satisfaction 27

Principal Component Analysis 31
- PCA on the determinants of a good kiss 31
- Varimax rotation 35
- Excluding participants from the new dataset 36

Models with variables from the PCA 36
- Models dim 1 ("technique") of a good kiss 36
- Models dim 2 ("sensory") of a good kiss 39

Participant sex and importance of the sensory component of a good kiss 42

This `R Markdown` document contains the code and analyses for **Watkins, C.D., Leongómez, J.D., Bovet, J., Zelazniewicz, A., Korbmacher, M., Corrêa Varella, M.A., Fernandez, A.M., Wagstaff, D., & Bolgan S. (2019).** ***National income inequality predicts cultural variation in mouth to mouth kissings***. *Manuscript in preparation.*

Preliminaries

Import packages

Loading data

```
load("Kissing_submitted_data_clean.RData")
```

Factors

```
db_sample$Sex <- as.factor(as.character(db_sample$Sex))
db_sample$RelStatus <- as.factor(as.character(db_sample$RelStatus))
```

Rescale variable GDP

```
## k$ instead of $
db_sample$GDP <- db_sample$GDP/1000
```

Excluding participants

```
db <- subset(db_sample, db_sample$Ex_Cross==0)
db$Country_Born <- droplevels(db$Country_Born)
db$Country_Res <- droplevels(db$Country_Res)
```

Individual differences in attitudes and behaviours related to kissing and intimacy

Mixed model

```
#Create database with only relevant columns, and *melting* kiss importance into one new
#column (Kiss_Imp), and one factor (Rel_phase)
db_Kiss_Imp <- melt(db, id.vars = c(1, 5, 7, 21), measure.vars = 27:28,
                    variable.name = "Rel_phase",
                    value.name = "Kiss_Imp")
db_Kiss_Imp$ID <- as.factor(db_Kiss_Imp$ID)
m_Kiss_phases <- lmer(Kiss_Imp ~ Rel_phase + Sex + SRA + Age +
                        Rel_phase:Sex + Rel_phase:SRA + Rel_phase:Age +
                        (1 | ID), data = db_Kiss_Imp)
anova(m_Kiss_phases, type = 3)
```

```
## Type III Analysis of Variance Table with Satterthwaite's method
##                Sum Sq Mean Sq NumDF  DenDF F value    Pr(>F)
## Rel_phase      4563.0  4563.0     1 1906.0 18.8364 1.499e-05 ***
## Sex            1223.5  1223.5     1 1905.3  5.0505  0.024733 *
## SRA            1898.8  1898.8     1 1907.1  7.8384  0.005166 **
## Age           12136.3 12136.3     1 1904.2 50.0992 2.047e-12 ***
## Rel_phase:Sex   111.0   111.0     1 1904.3  0.4581  0.498578
## Rel_phase:SRA    22.9    22.9     1 1906.1  0.0945  0.758561
## Rel_phase:Age  4959.6  4959.6     1 1903.2 20.4735 6.420e-06 ***
## ---
## Signif. codes:  0 '***' 0.001 '**' 0.01 '*' 0.05 '.' 0.1 ' ' 1
```

Follow-up tests

Importance of kissing by relationship phase

```
t.test(Kiss_Imp ~ Rel_phase, db_Kiss_Imp, conf.int=TRUE)
```

```
##
##  Welch Two Sample t-test
```

```
##
## data:  Kiss_Imp by Rel_phase
## t = -4.7615, df = 3669.8, p-value = 1.996e-06
## alternative hypothesis: true difference in means is not equal to 0
## 95 percent confidence interval:
##  -4.744784 -1.977005
## sample estimates:
## mean in group Kiss_Imp_Initial     mean in group Kiss_Imp_Est
##                        81.03848                       84.39938
```

```
group.CI(Kiss_Imp ~ Rel_phase, data = db_Kiss_Imp, ci = 0.95)
```

```
##          Rel_phase Kiss_Imp.upper Kiss_Imp.mean Kiss_Imp.lower
## 1 Kiss_Imp_Initial       82.11872      81.03848       79.95825
## 2     Kiss_Imp_Est       85.26505      84.39938       83.53370
```

```
cohen.d(Kiss_Imp ~ Rel_phase, data = db_Kiss_Imp)
```

```
##
## Cohen's d
##
## d estimate: -0.1535572 (negligible)
## 95 percent confidence interval:
##       lower       upper
## -0.21687839 -0.09023594
```

Importance of kissing by age

```
cor.test(db_Kiss_Imp$Kiss_Imp, db_Kiss_Imp$Age, conf.level = 0.95)
```

```
##
##  Pearson's product-moment correlation
##
## data:  db_Kiss_Imp$Kiss_Imp and db_Kiss_Imp$Age
## t = 8.3291, df = 3830, p-value < 2.2e-16
## alternative hypothesis: true correlation is not equal to 0
## 95 percent confidence interval:
##  0.1021507 0.1643524
## sample estimates:
##       cor
## 0.1333829
```

Importance of kissing by self-rated attractiveness

```
cor.test(db_Kiss_Imp$Kiss_Imp, db_Kiss_Imp$SRA, conf.level = 0.95)
```

```
##
##  Pearson's product-moment correlation
##
## data:  db_Kiss_Imp$Kiss_Imp and db_Kiss_Imp$SRA
## t = 3.3434, df = 3836, p-value = 0.0008354
## alternative hypothesis: true correlation is not equal to 0
## 95 percent confidence interval:
##  0.02230331 0.08539730
## sample estimates:
##        cor
```

```
## 0.05390411
```

Importance of kissing by sex

```r
t.test(Kiss_Imp ~ Sex, db_Kiss_Imp, conf.int=TRUE)
```

```
##
##  Welch Two Sample t-test
##
## data:  Kiss_Imp by Sex
## t = -1.6941, df = 1722.8, p-value = 0.09044
## alternative hypothesis: true difference in means is not equal to 0
## 95 percent confidence interval:
##  -3.0136557  0.2203602
## sample estimates:
## mean in group 1 mean in group 2
##        81.69523        83.09188
```

```r
group.CI(Kiss_Imp ~ Sex, data = db_Kiss_Imp, ci = 0.95)
```

```
##   Sex Kiss_Imp.upper Kiss_Imp.mean Kiss_Imp.lower
## 1   1       83.10347      81.69523       80.28699
## 2   2       83.88756      83.09188       82.29619
```

```r
cohen.d(Kiss_Imp ~ Sex, data = db_Kiss_Imp)
```

```
##
## Cohen's d
##
## d estimate: -0.06364984 (negligible)
## 95 percent confidence interval:
##        lower        upper
## -0.135122973  0.007823291
```

Multilevel models

Models Importance of kissing at initial phase

```r
m_Kiss_Imp_Initial_1 <- lmer(Kiss_Imp_Initial ~ HPP_9 +  (1|Country_Born), data=db)
summary(m_Kiss_Imp_Initial_1)
```

```
## Linear mixed model fit by REML. t-tests use Satterthwaite's method [
## lmerModLmerTest]
## Formula: Kiss_Imp_Initial ~ HPP_9 + (1 | Country_Born)
##    Data: db
##
## REML criterion at convergence: 17600.4
##
## Scaled residuals:
##     Min      1Q  Median      3Q     Max
## -3.6325 -0.3785  0.3276  0.6792  1.7596
##
## Random effects:
##  Groups       Name        Variance Std.Dev.
##  Country_Born (Intercept)  39.75    6.305
```

```
##  Residual                 547.09   23.390
## Number of obs: 1923, groups:  Country_Born, 13
##
## Fixed effects:
##             Estimate Std. Error     df t value Pr(>|t|)
## (Intercept)   78.581      1.931 11.085  40.692 2.01e-13 ***
## HPP_9         -5.431      2.481 11.033  -2.189    0.051 .
## ---
## Signif. codes:  0 '***' 0.001 '**' 0.01 '*' 0.05 '.' 0.1 ' ' 1
##
## Correlation of Fixed Effects:
##       (Intr)
## HPP_9 0.231
```

```
m_Kiss_Imp_Initial_2 <- lmer(Kiss_Imp_Initial ~ HPP_9 + GINI + GDP +  (1|Country_Born),
                             data=db)
summary(m_Kiss_Imp_Initial_2)
```

```
## Linear mixed model fit by REML. t-tests use Satterthwaite's method [
## lmerModLmerTest]
## Formula: Kiss_Imp_Initial ~ HPP_9 + GINI + GDP + (1 | Country_Born)
##    Data: db
##
## REML criterion at convergence: 17602.7
##
## Scaled residuals:
##     Min      1Q  Median      3Q     Max
## -3.6364 -0.3658  0.3285  0.6793  1.7742
##
## Random effects:
##  Groups       Name        Variance Std.Dev.
##  Country_Born (Intercept)  49.32    7.023
##  Residual                 547.09   23.390
## Number of obs: 1923, groups:  Country_Born, 13
##
## Fixed effects:
##             Estimate Std. Error       df t value Pr(>|t|)
## (Intercept) 82.36261   13.17784 10.07149   6.250 9.21e-05 ***
## HPP_9       -5.27464    4.60346  9.03243  -1.146    0.281
## GINI        -0.07956    0.25586  9.52070  -0.311    0.763
## GDP         -0.02328    0.21281  9.60737  -0.109    0.915
## ---
## Signif. codes:  0 '***' 0.001 '**' 0.01 '*' 0.05 '.' 0.1 ' ' 1
##
## Correlation of Fixed Effects:
##       (Intr) HPP_9  GINI
## HPP_9 -0.169
## GINI  -0.878 -0.184
## GDP   -0.659  0.711  0.255
```

```
m_Kiss_Imp_Initial_3 <- lmer(Kiss_Imp_Initial ~ HPP_9 + GINI + GDP + Sex + Age + SRA +
                               RelStatus + (1|Country_Born), data=db)
summary(m_Kiss_Imp_Initial_3)
```

```
## Linear mixed model fit by REML. t-tests use Satterthwaite's method [
```

```
## lmerModLmerTest]
## Formula:
## Kiss_Imp_Initial ~ HPP_9 + GINI + GDP + Sex + Age + SRA + RelStatus +
##     (1 | Country_Born)
##    Data: db
##
## REML criterion at convergence: 17330.4
##
## Scaled residuals:
##     Min      1Q  Median      3Q     Max
## -4.2768 -0.3532  0.3044  0.6713  2.0238
##
## Random effects:
##  Groups       Name        Variance Std.Dev.
##  Country_Born (Intercept)  63.56    7.973
##  Residual                 522.51   22.858
## Number of obs: 1903, groups:  Country_Born, 13
##
## Fixed effects:
##               Estimate Std. Error         df t value Pr(>|t|)
## (Intercept)   63.29020   14.92907   10.48970   4.239  0.00154 **
## HPP_9         -5.45674    5.15941    9.01652  -1.058  0.31774
## GINI          -0.08099    0.28580    9.37101  -0.283  0.78305
## GDP           -0.04368    0.23772    9.45177  -0.184  0.85809
## Sex2           1.55688    1.20752 1889.92086   1.289  0.19745
## Age            0.33345    0.04877 1894.04847   6.837 1.09e-11 ***
## SRA            2.06159    0.44695 1894.98591   4.613 4.24e-06 ***
## RelStatus2    -2.73401    1.23828 1887.25231  -2.208  0.02737 *
## ---
## Signif. codes:  0 '***' 0.001 '**' 0.01 '*' 0.05 '.' 0.1 ' ' 1
##
## Correlation of Fixed Effects:
##            (Intr) HPP_9  GINI   GDP    Sex2   Age    SRA
## HPP_9      -0.169
## GINI       -0.861 -0.185
## GDP        -0.646  0.714  0.248
## Sex2       -0.084  0.002  0.013 -0.001
## Age        -0.084 -0.005 -0.011 -0.022  0.130
## SRA        -0.146  0.001  0.003  0.017  0.005 -0.045
## RelStatus2 -0.024 -0.004 -0.026 -0.009  0.036  0.100  0.091
```

Summary Table Importance of kissing at initial phase

```
class(m_Kiss_Imp_Initial_1) <- "lmerMod"
class(m_Kiss_Imp_Initial_2) <- "lmerMod"
class(m_Kiss_Imp_Initial_3) <- "lmerMod"
stargazer(m_Kiss_Imp_Initial_1, m_Kiss_Imp_Initial_2, m_Kiss_Imp_Initial_3, type = "text",
          digits = 2, star.cutoffs = c(0.05, 0.01, 0.001),report=('vc*stp'))
```

```
##
## ===================================================
##                          Dependent variable:
##                    -------------------------------
##                           Kiss_Imp_Initial
```

```
##                        (1)        (2)       (3)
## --------------------------------------------------
## HPP_9                 -5.43*     -5.27     -5.46
##                       (2.48)    (4.60)     (5.16)
##                     t = -2.19  t = -1.15 t = -1.06
##                      p = 0.03  p = 0.26   p = 0.30
##
## GINI                             -0.08     -0.08
##                                 (0.26)     (0.29)
##                                t = -0.31 t = -0.28
##                                p = 0.76   p = 0.78
##
## GDP                              -0.02     -0.04
##                                 (0.21)     (0.24)
##                                t = -0.11 t = -0.18
##                                p = 0.92   p = 0.86
##
## Sex2                                        1.56
##                                            (1.21)
##                                           t = 1.29
##                                           p = 0.20
##
## Age                                       0.33***
##                                            (0.05)
##                                           t = 6.84
##                                           p = 0.00
##
## SRA                                       2.06***
##                                            (0.45)
##                                           t = 4.61
##                                          p = 0.0000
##
## RelStatus2                                 -2.73*
##                                            (1.24)
##                                          t = -2.21
##                                           p = 0.03
##
## Constant             78.58***  82.36***   63.29***
##                       (1.93)    (13.18)   (14.93)
##                     t = 40.69  t = 6.25   t = 4.24
##                      p = 0.00  p = 0.00  p = 0.0001
##
## --------------------------------------------------
## Observations          1,923      1,923     1,923
## Log Likelihood      -8,800.22  -8,801.37 -8,665.21
## Akaike Inf. Crit.   17,608.44  17,614.75 17,350.42
## Bayesian Inf. Crit. 17,630.69  17,648.12 17,405.93
## ==================================================
## Note:                 *p<0.05; **p<0.01; ***p<0.001
```

Models Importance of kissing at established phase

```
m_Kiss_Imp_Est_1 <- lmer(Kiss_Imp_Est ~ HPP_9 +  (1|Country_Born), data=db)
summary(m_Kiss_Imp_Est_1)
```

```
## Linear mixed model fit by REML. t-tests use Satterthwaite's method [
## lmerModLmerTest]
## Formula: Kiss_Imp_Est ~ HPP_9 + (1 | Country_Born)
##    Data: db
##
## REML criterion at convergence: 16830.2
##
## Scaled residuals:
##     Min      1Q  Median      3Q     Max
## -4.4110 -0.4432  0.2941  0.7110  1.1206
##
## Random effects:
##  Groups       Name        Variance Std.Dev.
##  Country_Born (Intercept)   9.189   3.031
##  Residual                 368.150  19.187
## Number of obs: 1923, groups:  Country_Born, 13
##
## Fixed effects:
##             Estimate Std. Error      df t value Pr(>|t|)
## (Intercept)  83.1983     1.0347 10.5117  80.411 5.24e-16 ***
## HPP_9        -0.4089     1.3260 10.2453  -0.308    0.764
## ---
## Signif. codes:  0 '***' 0.001 '**' 0.01 '*' 0.05 '.' 0.1 ' ' 1
##
## Correlation of Fixed Effects:
##       (Intr)
## HPP_9 0.238
```

```
m_Kiss_Imp_Est_2 <- lmer(Kiss_Imp_Est ~ HPP_9 + GINI + GDP +  (1|Country_Born), data=db)
summary(m_Kiss_Imp_Est_2)
```

```
## Linear mixed model fit by REML. t-tests use Satterthwaite's method [
## lmerModLmerTest]
## Formula: Kiss_Imp_Est ~ HPP_9 + GINI + GDP + (1 | Country_Born)
##    Data: db
##
## REML criterion at convergence: 16833.3
##
## Scaled residuals:
##     Min      1Q  Median      3Q     Max
## -4.4330 -0.4531  0.2971  0.7112  1.1336
##
## Random effects:
##  Groups       Name        Variance Std.Dev.
##  Country_Born (Intercept)   9.152   3.025
##  Residual                 368.170  19.188
## Number of obs: 1923, groups:  Country_Born, 13
##
## Fixed effects:
##             Estimate Std. Error       df t value Pr(>|t|)
## (Intercept) 74.57154    6.70793 11.68833  11.117 1.45e-07 ***
```

```
## HPP_9       -0.71842    2.23167  8.47045  -0.322    0.755
## GINI         0.17578    0.12721 10.02161   1.382    0.197
## GDP          0.05813    0.10631 10.40760   0.547    0.596
## ---
## Signif. codes:  0 '***' 0.001 '**' 0.01 '*' 0.05 '.' 0.1 ' ' 1
##
## Correlation of Fixed Effects:
##       (Intr) HPP_9  GINI
## HPP_9 -0.160
## GINI  -0.889 -0.177
## GDP   -0.681  0.695  0.303
```

```
m_Kiss_Imp_Est_3 <- lmer(Kiss_Imp_Est ~ HPP_9 + GINI + GDP + Sex + Age + SRA + RelStatus +
                           (1|Country_Born), data=db)
summary(m_Kiss_Imp_Est_3)
```

```
## Linear mixed model fit by REML. t-tests use Satterthwaite's method [
## lmerModLmerTest]
## Formula:
## Kiss_Imp_Est ~ HPP_9 + GINI + GDP + Sex + Age + SRA + RelStatus +
##     (1 | Country_Born)
##    Data: db
##
## REML criterion at convergence: 16615.6
##
## Scaled residuals:
##     Min      1Q  Median      3Q     Max
## -4.5110 -0.4540  0.3226  0.7296  1.3574
##
## Random effects:
##  Groups       Name        Variance Std.Dev.
##  Country_Born (Intercept)  10.92    3.305
##  Residual                 360.31   18.982
## Number of obs: 1903, groups:  Country_Born, 13
##
## Fixed effects:
##                Estimate Std. Error         df t value Pr(>|t|)
## (Intercept)    63.88747    7.44640   13.33167   8.580 8.59e-07 ***
## HPP_9          -0.65214    2.38204    8.26721  -0.274 0.790975
## GINI            0.18494    0.13495    9.52286   1.370 0.201973
## GDP             0.05275    0.11283    9.86952   0.468 0.650271
## Sex2            1.46302    1.00229 1894.43850   1.460 0.144544
## Age             0.13469    0.04033 1869.31370   3.340 0.000855 ***
## SRA             1.27718    0.37002 1849.39338   3.452 0.000570 ***
## RelStatus2     -1.24026    1.02825 1889.70419  -1.206 0.227893
## ---
## Signif. codes:  0 '***' 0.001 '**' 0.01 '*' 0.05 '.' 0.1 ' ' 1
##
## Correlation of Fixed Effects:
##            (Intr) HPP_9  GINI   GDP    Sex2   Age    SRA
## HPP_9      -0.156
## GINI       -0.843 -0.176
## GDP        -0.645  0.699  0.295
## Sex2       -0.139  0.007  0.020 -0.001
## Age        -0.141 -0.005 -0.018 -0.037  0.131
```

```
## SRA        -0.244  0.000  0.008  0.030  0.008 -0.044
## RelStatus2 -0.040 -0.008 -0.046 -0.017  0.035  0.103  0.089
```

Summary Table Importance of kissing at established phase

```
class(m_Kiss_Imp_Est_1) <- "lmerMod"
class(m_Kiss_Imp_Est_2) <- "lmerMod"
class(m_Kiss_Imp_Est_3) <- "lmerMod"
stargazer(m_Kiss_Imp_Est_1, m_Kiss_Imp_Est_2, m_Kiss_Imp_Est_3, type = "text", digits = 2,
          star.cutoffs = c(0.05, 0.01, 0.001),report=('vc*stp'))
```

```
##
## ===================================================
##                           Dependent variable:
##                      ------------------------------
##                               Kiss_Imp_Est
##                         (1)        (2)        (3)
## ---------------------------------------------------
## HPP_9                  -0.41      -0.72      -0.65
##                        (1.33)     (2.23)    (2.38)
##                      t = -0.31  t = -0.32  t = -0.27
##                       p = 0.76   p = 0.75  p = 0.79
##
## GINI                               0.18      0.18
##                                   (0.13)    (0.13)
##                                  t = 1.38  t = 1.37
##                                  p = 0.17  p = 0.18
##
## GDP                                0.06      0.05
##                                   (0.11)    (0.11)
##                                  t = 0.55  t = 0.47
##                                  p = 0.59  p = 0.65
##
## Sex2                                         1.46
##                                             (1.00)
##                                            t = 1.46
##                                            p = 0.15
##
## Age                                         0.13***
##                                             (0.04)
##                                            t = 3.34
##                                            p = 0.001
##
## SRA                                         1.28***
##                                             (0.37)
##                                            t = 3.45
##                                            p = 0.001
##
## RelStatus2                                   -1.24
##                                             (1.03)
##                                            t = -1.21
##                                            p = 0.23
##
## Constant              83.20***   74.57***  63.89***
```

```
##                          (1.03)      (6.71)    (7.45)
##                        t = 80.41  t = 11.12  t = 8.58
##                         p = 0.00   p = 0.00  p = 0.00
##
## ---------------------------------------------------
## Observations            1,923      1,923      1,903
## Log Likelihood      -8,415.11  -8,416.66  -8,307.80
## Akaike Inf. Crit.   16,838.22  16,845.32  16,635.61
## Bayesian Inf. Crit. 16,860.47  16,878.69  16,691.12
## ===================================================
## Note:                   *p<0.05; **p<0.01; ***p<0.001
```

Models frequency of kissing

```
m_Kiss_Freq_1 <- lmer(Kiss_Freq ~ HPP_9 +  (1|Country_Born), data=db)
summary(m_Kiss_Freq_1)
```

```
## Linear mixed model fit by REML. t-tests use Satterthwaite's method [
## lmerModLmerTest]
## Formula: Kiss_Freq ~ HPP_9 + (1 | Country_Born)
##    Data: db
##
## REML criterion at convergence: 16965.1
##
## Scaled residuals:
##     Min      1Q  Median      3Q     Max
## -3.9404 -0.5154  0.2101  0.9525  1.3183
##
## Random effects:
##  Groups       Name        Variance Std.Dev.
##  Country_Born (Intercept)   8.926   2.988
##  Residual                 417.699  20.438
## Number of obs: 1911, groups:  Country_Born, 13
##
## Fixed effects:
##             Estimate Std. Error      df t value Pr(>|t|)
## (Intercept)  77.2981     1.0449 10.6580  73.979  8.5e-16 ***
## HPP_9        -0.2087     1.3384 10.3289  -0.156    0.879
## ---
## Signif. codes:  0 '***' 0.001 '**' 0.01 '*' 0.05 '.' 0.1 ' ' 1
##
## Correlation of Fixed Effects:
##       (Intr)
## HPP_9 0.240
```

```
m_Kiss_Freq_2 <- lmer(Kiss_Freq ~ HPP_9 + GINI + GDP +  (1|Country_Born), data=db)
summary(m_Kiss_Freq_2)
```

```
## Linear mixed model fit by REML. t-tests use Satterthwaite's method [
## lmerModLmerTest]
## Formula: Kiss_Freq ~ HPP_9 + GINI + GDP + (1 | Country_Born)
##    Data: db
##
## REML criterion at convergence: 16961
##
```

```
## Scaled residuals:
##     Min      1Q  Median      3Q     Max
## -3.9324 -0.5055  0.2285  0.8956  1.3194
##
## Random effects:
##  Groups       Name        Variance Std.Dev.
##  Country_Born (Intercept)   3.263   1.806
##  Residual                 417.254  20.427
## Number of obs: 1911, groups:  Country_Born, 13
##
## Fixed effects:
##              Estimate Std. Error        df t value Pr(>|t|)
## (Intercept) 64.870163   5.434405 25.247951  11.937 7.03e-12 ***
## HPP_9       -2.931227   1.680493 12.723987  -1.744  0.10520
## GINI         0.321938   0.099885 18.935937   3.223  0.00449 **
## GDP         -0.008696   0.084362 21.088303  -0.103  0.91887
## ---
## Signif. codes:  0 '***' 0.001 '**' 0.01 '*' 0.05 '.' 0.1 ' ' 1
##
## Correlation of Fixed Effects:
##       (Intr) HPP_9  GINI
## HPP_9 -0.156
## GINI  -0.903 -0.160
## GDP   -0.710  0.679  0.368
```

```r
m_Kiss_Freq_3 <- lmer(Kiss_Freq ~ HPP_9 + GINI + GDP + Sex + Age + SRA + RelStatus +
                        (1|Country_Born), data=db)
summary(m_Kiss_Freq_3)
```

```
## Linear mixed model fit by REML. t-tests use Satterthwaite's method [
## lmerModLmerTest]
## Formula: Kiss_Freq ~ HPP_9 + GINI + GDP + Sex + Age + SRA + RelStatus +
##     (1 | Country_Born)
##    Data: db
##
## REML criterion at convergence: 16749.8
##
## Scaled residuals:
##     Min      1Q  Median      3Q     Max
## -3.9996 -0.5364  0.1993  0.8139  1.7649
##
## Random effects:
##  Groups       Name        Variance Std.Dev.
##  Country_Born (Intercept)   4.556   2.134
##  Residual                 406.448  20.161
## Number of obs: 1893, groups:  Country_Born, 13
##
## Fixed effects:
##                Estimate Std. Error         df t value Pr(>|t|)
## (Intercept)    56.35261    6.27396   28.33951   8.982 8.74e-10 ***
## HPP_9          -2.93631    1.82999   11.50528  -1.605  0.13567
## GINI            0.37143    0.10707   15.76772   3.469  0.00322 **
## GDP             0.01250    0.09031   17.18466   0.138  0.89154
## Sex2           -0.49241    1.06779 1884.13447  -0.461  0.64474
## Age            -0.01497    0.04266 1695.96554  -0.351  0.72563
```

```
## SRA            1.77752    0.39269 1718.04233   4.527 6.41e-06 ***
## RelStatus2    -4.55024    1.09637 1884.02067  -4.150 3.47e-05 ***
## ---
## Signif. codes:  0 '***' 0.001 '**' 0.01 '*' 0.05 '.' 0.1 ' ' 1
##
## Correlation of Fixed Effects:
##            (Intr) HPP_9  GINI   GDP    Sex2   Age    SRA
## HPP_9      -0.149
## GINI       -0.829 -0.161
## GDP        -0.648  0.684  0.350
## Sex2       -0.173  0.015  0.025  0.000
## Age        -0.182  0.004 -0.020 -0.046  0.133
## SRA        -0.311 -0.006  0.019  0.040  0.004 -0.044
## RelStatus2 -0.048 -0.015 -0.062 -0.024  0.034  0.103  0.086
```

Summary Table frequency of kissing

```r
class(m_Kiss_Freq_1) <- "lmerMod"
class(m_Kiss_Freq_2) <- "lmerMod"
class(m_Kiss_Freq_3) <- "lmerMod"
stargazer(m_Kiss_Freq_1, m_Kiss_Freq_2, m_Kiss_Freq_3, type = "text", digits = 2,
          star.cutoffs = c(0.05, 0.01, 0.001),report=('vc*stp'))
```

```
##
## ====================================================
##                            Dependent variable:
##                      -------------------------------
##                                 Kiss_Freq
##                         (1)        (2)       (3)
## ----------------------------------------------------
## HPP_9                  -0.21      -2.93     -2.94
##                        (1.34)     (1.68)     (1.83)
##                      t = -0.16  t = -1.74 t = -1.60
##                       p = 0.88  p = 0.09   p = 0.11
##
## GINI                             0.32**    0.37***
##                                  (0.10)     (0.11)
##                                 t = 3.22   t = 3.47
##                                 p = 0.002 p = 0.001
##
## GDP                              -0.01      0.01
##                                  (0.08)     (0.09)
##                                 t = -0.10  t = 0.14
##                                 p = 0.92   p = 0.89
##
## Sex2                                        -0.49
##                                             (1.07)
##                                           t = -0.46
##                                            p = 0.65
##
## Age                                         -0.01
##                                             (0.04)
##                                           t = -0.35
##                                            p = 0.73
```

```
##
## SRA                                       1.78***
##                                            (0.39)
##                                          t = 4.53
##                                         p = 0.0000
##
## RelStatus2                                -4.55***
##                                            (1.10)
##                                         t = -4.15
##                                         p = 0.0001
##
## Constant              77.30***  64.87***   56.35***
##                        (1.04)    (5.43)     (6.27)
##                      t = 73.98  t = 11.94  t = 8.98
##                       p = 0.00  p = 0.00   p = 0.00
##
## ---------------------------------------------------
## Observations           1,911      1,911     1,893
## Log Likelihood      -8,482.54  -8,480.50 -8,374.88
## Akaike Inf. Crit.   16,973.09  16,973.00 16,769.76
## Bayesian Inf. Crit. 16,995.31  17,006.33 16,825.22
## ===================================================
## Note:                  *p<0.05; **p<0.01; ***p<0.001
```

Models frequency of hugging

```
m_Hug_Freq_1 <- lmer(Hug_Freq ~ HPP_9 +  (1|Country_Born), data=db)
summary(m_Hug_Freq_1)
```

```
## Linear mixed model fit by REML. t-tests use Satterthwaite's method [
## lmerModLmerTest]
## Formula: Hug_Freq ~ HPP_9 + (1 | Country_Born)
##    Data: db
##
## REML criterion at convergence: 17252.5
##
## Scaled residuals:
##     Min      1Q  Median      3Q     Max
## -3.5114 -0.4525  0.2928  0.7982  1.2643
##
## Random effects:
##  Groups       Name        Variance Std.Dev.
##  Country_Born (Intercept)  20.47    4.524
##  Residual                 507.86   22.536
## Number of obs: 1901, groups:  Country_Born, 13
##
## Fixed effects:
##             Estimate Std. Error     df t value Pr(>|t|)
## (Intercept)   78.904      1.458 10.041  54.133 1.02e-13 ***
## HPP_9         -2.145      1.871  9.919  -1.146    0.279
## ---
## Signif. codes:  0 '***' 0.001 '**' 0.01 '*' 0.05 '.' 0.1 ' ' 1
##
## Correlation of Fixed Effects:
```

```
##       (Intr)
## HPP_9 0.235
```

```r
m_Hug_Freq_2 <- lmer(Hug_Freq ~ HPP_9 + GINI + GDP +  (1|Country_Born), data=db)
summary(m_Hug_Freq_2)
```

```
## Linear mixed model fit by REML. t-tests use Satterthwaite's method [
## lmerModLmerTest]
## Formula: Hug_Freq ~ HPP_9 + GINI + GDP + (1 | Country_Born)
##    Data: db
##
## REML criterion at convergence: 17247.9
##
## Scaled residuals:
##     Min      1Q  Median      3Q     Max
## -3.5201 -0.4593  0.2950  0.8225  1.2720
##
## Random effects:
##  Groups       Name        Variance Std.Dev.
##  Country_Born (Intercept)   7.646   2.765
##  Residual                 507.823  22.535
## Number of obs: 1901, groups:  Country_Born, 13
##
## Fixed effects:
##             Estimate Std. Error      df t value Pr(>|t|)
## (Intercept)  83.4926     6.8755 12.9229  12.144 1.93e-08 ***
## HPP_9        -8.2649     2.2201  8.1342  -3.723  0.00568 **
## GINI          0.1153     0.1287 10.4660   0.896  0.39043
## GDP          -0.3162     0.1080 11.1495  -2.929  0.01353 *
## ---
## Signif. codes:  0 '***' 0.001 '**' 0.01 '*' 0.05 '.' 0.1 ' ' 1
##
## Correlation of Fixed Effects:
##       (Intr) HPP_9  GINI
## HPP_9 -0.158
## GINI  -0.895 -0.170
## GDP   -0.693  0.687  0.331
```

```r
m_Hug_Freq_3 <- lmer(Hug_Freq ~ HPP_9 + GINI + GDP + Sex + Age + SRA + RelStatus +
                       (1|Country_Born), data=db)
summary(m_Hug_Freq_3)
```

```
## Linear mixed model fit by REML. t-tests use Satterthwaite's method [
## lmerModLmerTest]
## Formula: Hug_Freq ~ HPP_9 + GINI + GDP + Sex + Age + SRA + RelStatus +
##     (1 | Country_Born)
##    Data: db
##
## REML criterion at convergence: 17040.5
##
## Scaled residuals:
##     Min      1Q  Median      3Q     Max
## -3.6179 -0.4660  0.2901  0.7989  1.4683
##
## Random effects:
```

```
##  Groups       Name        Variance Std.Dev.
##  Country_Born (Intercept)   8.809   2.968
##  Residual                 499.356  22.346
## Number of obs: 1882, groups:  Country_Born, 13
##
## Fixed effects:
##              Estimate Std. Error         df t value Pr(>|t|)
## (Intercept)  82.80213    7.63274   15.67166  10.848 1.08e-08 ***
## HPP_9        -8.37423    2.32457    7.79606  -3.602  0.00726 **
## GINI          0.16518    0.13387    9.76270   1.234  0.24612
## GDP          -0.30289    0.11242   10.37063  -2.694  0.02187 *
## Sex2          2.11901    1.18716 1873.92621   1.785  0.07443 .
## Age          -0.08969    0.04755 1764.44564  -1.886  0.05943 .
## SRA           0.26147    0.43925 1751.82663   0.595  0.55174
## RelStatus2   -5.84688    1.21810 1870.45845  -4.800 1.71e-06 ***
## ---
## Signif. codes:  0 '***' 0.001 '**' 0.01 '*' 0.05 '.' 0.1 ' ' 1
##
## Correlation of Fixed Effects:
##            (Intr) HPP_9  GINI   GDP    Sex2   Age    SRA
## HPP_9      -0.151
## GINI       -0.835 -0.168
## GDP        -0.646  0.690  0.324
## Sex2       -0.160  0.011  0.022  0.000
## Age        -0.162 -0.002 -0.021 -0.045  0.133
## SRA        -0.286 -0.004  0.015  0.036  0.008 -0.045
## RelStatus2 -0.044 -0.014 -0.055 -0.023  0.034  0.102  0.089
```

Summary Table frequency of hugging

```
class(m_Hug_Freq_1) <- "lmerMod"
class(m_Hug_Freq_2) <- "lmerMod"
class(m_Hug_Freq_3) <- "lmerMod"
stargazer(m_Hug_Freq_1, m_Hug_Freq_2, m_Hug_Freq_3, type = "text", digits = 2,
          star.cutoffs = c(0.05, 0.01, 0.001),report=('vc*stp'))
```

```
##
## ===================================================
##                           Dependent variable:
##                      ------------------------------
##                                 Hug_Freq
##                         (1)       (2)        (3)
## ---------------------------------------------------
## HPP_9                  -2.14    -8.26***   -8.37***
##                       (1.87)     (2.22)     (2.32)
##                      t = -1.15 t = -3.72  t = -3.60
##                      p = 0.26  p = 0.0002 p = 0.0004
##
## GINI                             0.12       0.17
##                                 (0.13)     (0.13)
##                                t = 0.90   t = 1.23
##                                p = 0.38   p = 0.22
##
## GDP                            -0.32**    -0.30**
```

```
##                                 (0.11)     (0.11)
##                               t = -2.93  t = -2.69
##                               p = 0.004   p = 0.01
##
## Sex2                                       2.12
##                                           (1.19)
##                                          t = 1.78
##                                          p = 0.08
##
## Age                                       -0.09
##                                           (0.05)
##                                         t = -1.89
##                                          p = 0.06
##
## SRA                                        0.26
##                                           (0.44)
##                                          t = 0.60
##                                          p = 0.56
##
## RelStatus2                               -5.85***
##                                           (1.22)
##                                         t = -4.80
##                                         p = 0.0000
##
## Constant             78.90***   83.49***   82.80***
##                       (1.46)     (6.88)     (7.63)
##                     t = 54.13 t = 12.14  t = 10.85
##                     p = 0.00   p = 0.00   p = 0.00
##
## ---------------------------------------------------
## Observations          1,901      1,901      1,882
## Log Likelihood      -8,626.26 -8,623.96  -8,520.25
## Akaike Inf. Crit.   17,260.53 17,259.93  17,060.51
## Bayesian Inf. Crit. 17,282.73 17,293.23  17,115.91
## ===================================================
## Note:                 *p<0.05; **p<0.01; ***p<0.001
```

Models sex frequency

```
m_Sex_Freq_1 <- lmer(Sex_Freq ~ HPP_9 +  (1|Country_Born), data=db)
summary(m_Sex_Freq_1)
```

```
## Linear mixed model fit by REML. t-tests use Satterthwaite's method [
## lmerModLmerTest]
## Formula: Sex_Freq ~ HPP_9 + (1 | Country_Born)
##    Data: db
##
## REML criterion at convergence: 17195.8
##
## Scaled residuals:
##     Min      1Q  Median      3Q     Max
## -2.8511 -0.6237  0.1301  0.7038  1.9140
##
## Random effects:
```

```
##  Groups       Name        Variance Std.Dev.
##  Country_Born (Intercept)  20.77    4.557
##  Residual                 570.18   23.878
## Number of obs: 1871, groups:  Country_Born, 13
##
## Fixed effects:
##             Estimate Std. Error     df t value Pr(>|t|)
## (Intercept)   62.823      1.490 10.849   42.17 2.23e-13 ***
## HPP_9         -2.449      1.913 10.704   -1.28    0.228
## ---
## Signif. codes:  0 '***' 0.001 '**' 0.01 '*' 0.05 '.' 0.1 ' ' 1
##
## Correlation of Fixed Effects:
##       (Intr)
## HPP_9 0.240
```

```
m_Sex_Freq_2 <- lmer(Sex_Freq ~ HPP_9 + GINI + GDP +  (1|Country_Born), data=db)
summary(m_Sex_Freq_2)
```

```
## Linear mixed model fit by REML. t-tests use Satterthwaite's method [
## lmerModLmerTest]
## Formula: Sex_Freq ~ HPP_9 + GINI + GDP + (1 | Country_Born)
##    Data: db
##
## REML criterion at convergence: 17197.1
##
## Scaled residuals:
##     Min      1Q  Median      3Q     Max
## -2.8643 -0.6241  0.1296  0.6951  1.9076
##
## Random effects:
##  Groups       Name        Variance Std.Dev.
##  Country_Born (Intercept)  19.32    4.396
##  Residual                 570.27   23.880
## Number of obs: 1871, groups:  Country_Born, 13
##
## Fixed effects:
##              Estimate Std. Error        df t value Pr(>|t|)
## (Intercept) 52.877150   9.295473 10.749651   5.688 0.000153 ***
## HPP_9       -4.446613   3.129108  8.248730  -1.421 0.191984
## GINI         0.256854   0.177313  9.459660   1.449 0.179773
## GDP         -0.006388   0.147674  9.669816  -0.043 0.966375
## ---
## Signif. codes:  0 '***' 0.001 '**' 0.01 '*' 0.05 '.' 0.1 ' ' 1
##
## Correlation of Fixed Effects:
##       (Intr) HPP_9  GINI
## HPP_9 -0.158
## GINI  -0.886 -0.182
## GDP   -0.675  0.696  0.291
```

```
m_Sex_Freq_3 <- lmer(Sex_Freq ~ HPP_9 + GINI + GDP + Sex + Age + SRA +
                       RelStatus +  (1|Country_Born), data=db)
summary(m_Sex_Freq_3)
```

```
## Linear mixed model fit by REML. t-tests use Satterthwaite's method [
## lmerModLmerTest]
## Formula: Sex_Freq ~ HPP_9 + GINI + GDP + Sex + Age + SRA + RelStatus +
##     (1 | Country_Born)
##    Data: db
##
## REML criterion at convergence: 17007.3
##
## Scaled residuals:
##     Min      1Q  Median      3Q     Max
## -3.1619 -0.5833  0.1027  0.7246  2.1157
##
## Random effects:
##  Groups       Name        Variance Std.Dev.
##  Country_Born (Intercept)  28.24    5.314
##  Residual                 560.65   23.678
## Number of obs: 1854, groups:  Country_Born, 13
##
## Fixed effects:
##               Estimate Std. Error         df t value Pr(>|t|)
## (Intercept)   37.67314   11.01660   11.84344   3.420  0.00517 **
## HPP_9         -4.30669    3.64168    8.47751  -1.183  0.26907
## GINI           0.27591    0.20439    9.32497   1.350  0.20889
## GDP            0.03218    0.17024    9.49292   0.189  0.85405
## Sex2           1.30784    1.26628 1844.26255   1.033  0.30183
## Age           -0.03880    0.05087 1842.24945  -0.763  0.44565
## SRA            2.92362    0.47110 1835.94367   6.206  6.7e-10 ***
## RelStatus2    -0.03250    1.30943 1839.52677  -0.025  0.98020
## ---
## Signif. codes:  0 '***' 0.001 '**' 0.01 '*' 0.05 '.' 0.1 ' ' 1
##
## Correlation of Fixed Effects:
##            (Intr) HPP_9  GINI   GDP    Sex2   Age    SRA
## HPP_9      -0.158
## GINI       -0.851 -0.182
## GDP        -0.646  0.703  0.276
## Sex2       -0.116  0.006  0.017 -0.002
## Age        -0.118 -0.005 -0.017 -0.031  0.128
## SRA        -0.208  0.000  0.004  0.025 -0.001 -0.044
## RelStatus2 -0.032 -0.008 -0.034 -0.016  0.040  0.088  0.080
```

Summary Table sex frequency

```
class(m_Sex_Freq_1) <- "lmerMod"
class(m_Sex_Freq_2) <- "lmerMod"
class(m_Sex_Freq_3) <- "lmerMod"
stargazer(m_Sex_Freq_1, m_Sex_Freq_2, m_Sex_Freq_3, type = "text", digits = 2,
          star.cutoffs = c(0.05, 0.01, 0.001),report=('vc*stp'))
```

```
##
## ===================================================
##                          Dependent variable:
##                     -------------------------------
##                                Sex_Freq
```

```
##                          (1)        (2)        (3)
## ---------------------------------------------------
## HPP_9                  -2.45      -4.45      -4.31
##                        (1.91)     (3.13)     (3.64)
##                      t = -1.28  t = -1.42  t = -1.18
##                       p = 0.21   p = 0.16  p = 0.24
##
## GINI                              0.26       0.28
##                                  (0.18)     (0.20)
##                                 t = 1.45   t = 1.35
##                                 p = 0.15   p = 0.18
##
## GDP                              -0.01       0.03
##                                  (0.15)     (0.17)
##                                t = -0.04   t = 0.19
##                                 p = 0.97   p = 0.86
##
## Sex2                                         1.31
##                                             (1.27)
##                                            t = 1.03
##                                            p = 0.31
##
## Age                                         -0.04
##                                             (0.05)
##                                            t = -0.76
##                                            p = 0.45
##
## SRA                                         2.92***
##                                             (0.47)
##                                            t = 6.21
##                                            p = 0.00
##
## RelStatus2                                  -0.03
##                                             (1.31)
##                                            t = -0.02
##                                            p = 0.99
##
## Constant              62.82***   52.88***  37.67***
##                        (1.49)     (9.30)    (11.02)
##                      t = 42.17   t = 5.69  t = 3.42
##                       p = 0.00   p = 0.00  p = 0.001
##
## ---------------------------------------------------
## Observations           1,871      1,871      1,854
## Log Likelihood      -8,597.88  -8,598.57  -8,503.67
## Akaike Inf. Crit.   17,203.75  17,209.13  17,027.34
## Bayesian Inf. Crit. 17,225.89  17,242.34  17,082.60
## ===================================================
## Note:                 *p<0.05; **p<0.01; ***p<0.001
```

Models kissing satisfaction

```r
m_Kiss_Satis_1 <- lmer(Kiss_Satis ~ HPP_9 +  (1|Country_Born), data=db)
summary(m_Kiss_Satis_1)
```

```
## Linear mixed model fit by REML. t-tests use Satterthwaite's method [
## lmerModLmerTest]
## Formula: Kiss_Satis ~ HPP_9 + (1 | Country_Born)
##    Data: db
##
## REML criterion at convergence: 17220.8
##
## Scaled residuals:
##     Min      1Q  Median      3Q     Max
## -3.5482 -0.4766  0.2765  0.8655  0.9639
##
## Random effects:
##  Groups       Name        Variance Std.Dev.
##  Country_Born (Intercept)   1.81    1.345
##  Residual                 508.00   22.539
## Number of obs: 1899, groups:  Country_Born, 13
##
## Fixed effects:
##             Estimate Std. Error      df t value Pr(>|t|)
## (Intercept)  79.5515     0.7332  5.6412 108.505 1.39e-10 ***
## HPP_9         0.5273     0.9144  4.2457   0.577    0.593
## ---
## Signif. codes:  0 '***' 0.001 '**' 0.01 '*' 0.05 '.' 0.1 ' ' 1
##
## Correlation of Fixed Effects:
##       (Intr)
## HPP_9 0.292
```

```r
m_Kiss_Satis_2 <- lmer(Kiss_Satis ~ HPP_9 + GINI + GDP +  (1|Country_Born), data=db)
```

```
## singular fit
```

```r
# using blmer to deal with singularity
m_Kiss_Satis_2 <- blmer(Kiss_Satis ~ HPP_9 + GINI + GDP +  (1|Country_Born), data=db)
summary(m_Kiss_Satis_2)
```

```
## Cov prior  : Country_Born ~ wishart(df = 3.5, scale = Inf, posterior.scale = cov, common.scale = TRU
## Prior dev  : 7.5972
##
## Linear mixed model fit by REML ['blmerMod']
## Formula: Kiss_Satis ~ HPP_9 + GINI + GDP + (1 | Country_Born)
##    Data: db
##
## REML criterion at convergence: 17222.3
##
## Scaled residuals:
##     Min      1Q  Median      3Q     Max
## -3.6351 -0.4834  0.2981  0.8141  1.0420
##
## Random effects:
##  Groups       Name        Variance Std.Dev.
##  Country_Born (Intercept)   3.201   1.789
```

```
##  Residual                 506.836  22.513
## Number of obs: 1899, groups:  Country_Born, 13
##
## Fixed effects:
##             Estimate Std. Error t value
## (Intercept) 71.74106    5.79502  12.380
## HPP_9       -1.35877    1.76694  -0.769
## GINI         0.20109    0.10596   1.898
## GDP         -0.01294    0.08967  -0.144
##
## Correlation of Fixed Effects:
##       (Intr) HPP_9  GINI
## HPP_9 -0.155
## GINI  -0.905 -0.157
## GDP   -0.715  0.677  0.379
```

```
m_Kiss_Satis_3 <- lmer(Kiss_Satis ~ HPP_9 + GINI + GDP + Sex + Age + SRA + RelStatus +
                         (1|Country_Born), data=db)
```

```
## singular fit
```

```
# using blmer to deal with singularity
m_Kiss_Satis_3 <- blmer(Kiss_Satis ~ HPP_9 + GINI + GDP + Sex + Age + SRA + RelStatus +
                         (1|Country_Born), data=db)
summary(m_Kiss_Satis_3)
```

```
## Cov prior  : Country_Born ~ wishart(df = 3.5, scale = Inf, posterior.scale = cov, common.scale = TRUI
## Prior dev  : 7.5971
##
## Linear mixed model fit by REML ['blmerMod']
## Formula: Kiss_Satis ~ HPP_9 + GINI + GDP + Sex + Age + SRA + RelStatus +
##     (1 | Country_Born)
##    Data: db
##
## REML criterion at convergence: 17009.9
##
## Scaled residuals:
##     Min      1Q  Median      3Q     Max
## -3.8268 -0.5291  0.2836  0.7911  1.3744
##
## Random effects:
##  Groups       Name        Variance Std.Dev.
##  Country_Born (Intercept)   3.125   1.768
##  Residual                 494.876  22.246
## Number of obs: 1881, groups:  Country_Born, 13
##
## Fixed effects:
##             Estimate Std. Error t value
## (Intercept) 68.79631    6.38427  10.776
## HPP_9       -1.34666    1.75668  -0.767
## GINI         0.24529    0.10529   2.330
## GDP          0.02438    0.08934   0.273
## Sex2        -0.52802    1.18126  -0.447
## Age         -0.17270    0.04718  -3.660
## SRA          1.52023    0.43403   3.503
```

```
## RelStatus2  -4.01977    1.21243  -3.315
##
## Correlation of Fixed Effects:
##            (Intr) HPP_9  GINI   GDP    Sex2   Age    SRA
## HPP_9      -0.148
## GINI       -0.821 -0.151
## GDP        -0.650  0.678  0.382
## Sex2       -0.189  0.022  0.027  0.003
## Age        -0.205  0.016 -0.019 -0.048  0.133
## SRA        -0.340 -0.010  0.025  0.045  0.005 -0.037
## RelStatus2 -0.050 -0.018 -0.071 -0.026  0.036  0.105  0.083
```

Summary Table kissing satisfaction

```r
class(m_Kiss_Satis_1) <- "lmerMod"
class(m_Kiss_Satis_2) <- "lmerMod"
class(m_Kiss_Satis_3) <- "lmerMod"
stargazer(m_Kiss_Satis_1, m_Kiss_Satis_2, m_Kiss_Satis_3, type = "text", digits = 2,
          star.cutoffs = c(0.05, 0.01, 0.001),report=('vc*stp'))
```

```
##
## ===================================================
##                           Dependent variable:
##                       -------------------------------
##                                Kiss_Satis
##                          (1)        (2)       (3)
## ---------------------------------------------------
## HPP_9                    0.53      -1.36     -1.35
##                         (0.91)    (1.77)     (1.76)
##                       t = 0.58  t = -0.77 t = -0.77
##                       p = 0.57  p = 0.45   p = 0.45
##
## GINI                               0.20      0.25*
##                                   (0.11)     (0.11)
##                                 t = 1.90   t = 2.33
##                                 p = 0.06   p = 0.02
##
## GDP                                -0.01      0.02
##                                   (0.09)     (0.09)
##                                 t = -0.14  t = 0.27
##                                 p = 0.89   p = 0.79
##
## Sex2                                         -0.53
##                                              (1.18)
##                                            t = -0.45
##                                             p = 0.66
##
## Age                                        -0.17***
##                                              (0.05)
##                                            t = -3.66
##                                            p = 0.0003
##
## SRA                                         1.52***
##                                              (0.43)
```

```
##                                          t = 3.50
##                                         p = 0.0005
##
## RelStatus2                               -4.02***
##                                           (1.21)
##                                         t = -3.32
##                                         p = 0.001
##
## Constant              79.55***  71.74***   68.80***
##                        (0.73)    (5.80)     (6.38)
##                     t = 108.51 t = 12.38 t = 10.78
##                      p = 0.00  p = 0.00   p = 0.00
##
## ---------------------------------------------------
## Observations           1,899      1,899     1,881
## Log Likelihood      -8,610.40  -8,611.14 -8,504.96
## Akaike Inf. Crit.   17,228.81  17,234.27 17,029.93
## Bayesian Inf. Crit. 17,251.00  17,267.57 17,085.32
## ===================================================
## Note:                  *p<0.05; **p<0.01; ***p<0.001
```

Models Huging satisfaction

```
m_Hug_Satis_1 <- lmer(Hug_Satis ~ HPP_9 +  (1|Country_Born), data=db)
summary(m_Hug_Satis_1)
```

```
## Linear mixed model fit by REML. t-tests use Satterthwaite's method [
## lmerModLmerTest]
## Formula: Hug_Satis ~ HPP_9 + (1 | Country_Born)
##    Data: db
##
## REML criterion at convergence: 16982.9
##
## Scaled residuals:
##     Min      1Q  Median      3Q     Max
## -3.8601 -0.4878  0.3434  0.8104  0.9897
##
## Random effects:
##  Groups       Name        Variance Std.Dev.
##  Country_Born (Intercept)   2.716   1.648
##  Residual                 458.419  21.411
## Number of obs: 1894, groups:  Country_Born, 13
##
## Fixed effects:
##             Estimate Std. Error      df t value Pr(>|t|)
## (Intercept)  80.8966     0.7679  7.7876 105.353 1.45e-13 ***
## HPP_9        -0.8078     0.9695  6.5643  -0.833    0.434
## ---
## Signif. codes:  0 '***' 0.001 '**' 0.01 '*' 0.05 '.' 0.1 ' ' 1
##
## Correlation of Fixed Effects:
##       (Intr)
## HPP_9 0.270
```

```r
m_Hug_Satis_2 <- lmer(Hug_Satis ~ HPP_9 + GINI + GDP +  (1|Country_Born), data=db)
summary(m_Hug_Satis_2)
```

```
## Linear mixed model fit by REML. t-tests use Satterthwaite's method [
## lmerModLmerTest]
## Formula: Hug_Satis ~ HPP_9 + GINI + GDP + (1 | Country_Born)
##    Data: db
##
## REML criterion at convergence: 16985.2
##
## Scaled residuals:
##     Min      1Q  Median      3Q     Max
## -3.8605 -0.4832  0.3448  0.8121  1.0985
##
## Random effects:
##  Groups       Name        Variance Std.Dev.
##  Country_Born (Intercept)   2.651   1.628
##  Residual                 458.016  21.401
## Number of obs: 1894, groups:  Country_Born, 13
##
## Fixed effects:
##             Estimate Std. Error       df t value Pr(>|t|)
## (Intercept) 70.68366    5.45116 19.82728  12.967 3.85e-11 ***
## HPP_9       -0.80558    1.65067  8.83857  -0.488   0.6374
## GINI         0.18917    0.09942 14.36119   1.903   0.0773 .
## GDP          0.09081    0.08416 16.30127   1.079   0.2963
## ---
## Signif. codes:  0 '***' 0.001 '**' 0.01 '*' 0.05 '.' 0.1 ' ' 1
##
## Correlation of Fixed Effects:
##       (Intr) HPP_9  GINI
## HPP_9 -0.153
## GINI  -0.906 -0.158
## GDP   -0.717  0.675  0.384
```

```r
m_Hug_Satis_3 <- lmer(Hug_Satis ~ HPP_9 + GINI + GDP + Sex + Age + SRA + RelStatus +
                        (1|Country_Born), data=db)
summary(m_Hug_Satis_3)
```

```
## Linear mixed model fit by REML. t-tests use Satterthwaite's method [
## lmerModLmerTest]
## Formula: Hug_Satis ~ HPP_9 + GINI + GDP + Sex + Age + SRA + RelStatus +
##     (1 | Country_Born)
##    Data: db
##
## REML criterion at convergence: 16726.6
##
## Scaled residuals:
##     Min      1Q  Median      3Q     Max
## -3.9595 -0.4612  0.3180  0.7079  1.6734
##
## Random effects:
##  Groups       Name        Variance Std.Dev.
##  Country_Born (Intercept)   6.342   2.518
```

```
##  Residual                 434.827  20.852
## Number of obs: 1876, groups:  Country_Born, 13
##
## Fixed effects:
##              Estimate Std. Error        df t value Pr(>|t|)
## (Intercept)   70.5672     6.8489   22.0618  10.303 6.78e-10 ***
## HPP_9         -1.1925     2.0469   10.0547  -0.583  0.57301
## GINI           0.2612     0.1185   13.0155   2.204  0.04613 *
## GDP            0.1236     0.0997   13.9581   1.240  0.23533
## Sex2           0.5642     1.1121 1867.8151   0.507  0.61198
## Age           -0.2636     0.0445 1746.1033  -5.924 3.78e-09 ***
## SRA            1.2782     0.4101 1723.3012   3.117  0.00186 **
## RelStatus2    -7.4947     1.1400 1866.0397  -6.574 6.33e-11 ***
## ---
## Signif. codes:  0 '***' 0.001 '**' 0.01 '*' 0.05 '.' 0.1 ' ' 1
##
## Correlation of Fixed Effects:
##            (Intr) HPP_9  GINI   GDP    Sex2   Age    SRA
## HPP_9      -0.150
## GINI       -0.831 -0.166
## GDP        -0.647  0.687  0.335
## Sex2       -0.167  0.017  0.023  0.003
## Age        -0.172  0.002 -0.021 -0.043  0.132
## SRA        -0.300 -0.004  0.019  0.040  0.006 -0.044
## RelStatus2 -0.047 -0.014 -0.056 -0.022  0.031  0.099  0.089
```

Summary Table Huging satisfaction

```r
class(m_Hug_Satis_1) <- "lmerMod"
class(m_Hug_Satis_2) <- "lmerMod"
class(m_Hug_Satis_3) <- "lmerMod"
stargazer(m_Hug_Satis_1, m_Hug_Satis_2, m_Hug_Satis_3, type = "text", digits = 2,
          star.cutoffs = c(0.05, 0.01, 0.001),report=('vc*stp'))
```

```
##
## ===================================================
##                           Dependent variable:
##                     -------------------------------
##                                Hug_Satis
##                         (1)        (2)       (3)
## ---------------------------------------------------
## HPP_9                  -0.81      -0.81     -1.19
##                       (0.97)     (1.65)    (2.05)
##                     t = -0.83  t = -0.49 t = -0.58
##                     p = 0.41   p = 0.63  p = 0.57
##
## GINI                              0.19      0.26*
##                                  (0.10)    (0.12)
##                                t = 1.90  t = 2.20
##                                p = 0.06  p = 0.03
##
## GDP                               0.09      0.12
##                                  (0.08)    (0.10)
##                                t = 1.08  t = 1.24
```

```
##                                  p = 0.29  p = 0.22
##
## Sex2                                        0.56
##                                            (1.11)
##                                           t = 0.51
##                                           p = 0.62
##
## Age                                       -0.26***
##                                            (0.04)
##                                           t = -5.92
##                                           p = 0.00
##
## SRA                                        1.28**
##                                            (0.41)
##                                           t = 3.12
##                                           p = 0.002
##
## RelStatus2                                -7.49***
##                                            (1.14)
##                                           t = -6.57
##                                           p = 0.00
##
## Constant               80.90***   70.68***  70.57***
##                         (0.77)     (5.45)    (6.85)
##                       t = 105.35  t = 12.97 t = 10.30
##                        p = 0.00   p = 0.00  p = 0.00
##
## ---------------------------------------------------
## Observations             1,894      1,894     1,876
## Log Likelihood         -8,491.45  -8,492.59 -8,363.31
## Akaike Inf. Crit.      16,990.90  16,997.19 16,746.63
## Bayesian Inf. Crit.    17,013.09  17,030.47 16,802.00
## ===================================================
## Note:                   *p<0.05; **p<0.01; ***p<0.001
```

Models Sex satisfaction

```
m_Sex_Satis_1 <- lmer(Sex_Satis ~ HPP_9 +  (1|Country_Born), data=db)
summary(m_Sex_Satis_1)
```

```
## Linear mixed model fit by REML. t-tests use Satterthwaite's method [
## lmerModLmerTest]
## Formula: Sex_Satis ~ HPP_9 + (1 | Country_Born)
##    Data: db
##
## REML criterion at convergence: 17405.5
##
## Scaled residuals:
##     Min      1Q  Median      3Q     Max
## -2.8608 -0.7525  0.2394  0.9104  1.2734
##
## Random effects:
##  Groups       Name        Variance Std.Dev.
##  Country_Born (Intercept)   8.73    2.955
```

```
##  Residual                 687.11   26.213
## Number of obs: 1857, groups:  Country_Born, 13
##
## Fixed effects:
##             Estimate Std. Error      df t value Pr(>|t|)
## (Intercept)  71.1731     1.1463 10.4465  62.091 9.29e-15 ***
## HPP_9        -0.8093     1.4634  9.7494  -0.553    0.593
## ---
## Signif. codes:  0 '***' 0.001 '**' 0.01 '*' 0.05 '.' 0.1 ' ' 1
##
## Correlation of Fixed Effects:
##       (Intr)
## HPP_9 0.253
```

```
m_Sex_Satis_2 <- lmer(Sex_Satis ~ HPP_9 + GINI + GDP +  (1|Country_Born), data=db)
summary(m_Sex_Satis_2)
```

```
## Linear mixed model fit by REML. t-tests use Satterthwaite's method [
## lmerModLmerTest]
## Formula: Sex_Satis ~ HPP_9 + GINI + GDP + (1 | Country_Born)
##    Data: db
##
## REML criterion at convergence: 17404.5
##
## Scaled residuals:
##     Min      1Q  Median      3Q     Max
## -2.8745 -0.7230  0.2344  0.9028  1.2618
##
## Random effects:
##  Groups       Name        Variance Std.Dev.
##  Country_Born (Intercept)   2.186   1.479
##  Residual                 687.580  26.222
## Number of obs: 1857, groups:  Country_Born, 13
##
## Fixed effects:
##             Estimate Std. Error       df t value Pr(>|t|)
## (Intercept) 61.90001    6.35380 14.63702   9.742 8.74e-08 ***
## HPP_9       -3.80320    1.83459  4.98673  -2.073   0.0930 .
## GINI         0.27143    0.11431 10.02704   2.375   0.0389 *
## GDP         -0.05780    0.09686 11.76330  -0.597   0.5620
## ---
## Signif. codes:  0 '***' 0.001 '**' 0.01 '*' 0.05 '.' 0.1 ' ' 1
##
## Correlation of Fixed Effects:
##       (Intr) HPP_9  GINI
## HPP_9 -0.146
## GINI  -0.913 -0.153
## GDP   -0.732  0.666  0.419
```

```
m_Sex_Satis_3 <- lmer(Sex_Satis ~ HPP_9 + GINI + GDP + Sex + Age + SRA + RelStatus +
                        (1|Country_Born), data=db)
summary(m_Sex_Satis_3)
```

```
## Linear mixed model fit by REML. t-tests use Satterthwaite's method [
## lmerModLmerTest]
```

```
## Formula: Sex_Satis ~ HPP_9 + GINI + GDP + Sex + Age + SRA + RelStatus +
##     (1 | Country_Born)
##    Data: db
##
## REML criterion at convergence: 17222.8
##
## Scaled residuals:
##     Min      1Q  Median      3Q     Max
## -2.9548 -0.6874  0.2321  0.8636  1.5789
##
## Random effects:
##  Groups       Name        Variance Std.Dev.
##  Country_Born (Intercept)   1.561   1.249
##  Residual                 681.588  26.107
## Number of obs: 1840, groups:  Country_Born, 13
##
## Fixed effects:
##              Estimate Std. Error         df t value Pr(>|t|)
## (Intercept)   52.28465    7.01249   26.89481   7.456 5.21e-08 ***
## HPP_9         -3.87181    1.76286    4.88616  -2.196   0.0807 .
## GINI           0.30822    0.11120   10.93000   2.772   0.0183 *
## GDP           -0.03495    0.09470   12.92555  -0.369   0.7181
## Sex2           5.53268    1.39783 1821.13421   3.958 7.85e-05 ***
## Age           -0.07660    0.05507  583.34351  -1.391   0.1648
## SRA            1.27481    0.51540 1014.59888   2.473   0.0135 *
## RelStatus2    -0.26954    1.44780 1828.69326  -0.186   0.8523
## ---
## Signif. codes:  0 '***' 0.001 '**' 0.01 '*' 0.05 '.' 0.1 ' ' 1
##
## Correlation of Fixed Effects:
##            (Intr) HPP_9  GINI   GDP    Sex2   Age    SRA
## HPP_9      -0.137
## GINI       -0.819 -0.141
## GDP        -0.658  0.664  0.435
## Sex2       -0.202  0.027  0.032  0.004
## Age        -0.229  0.043 -0.015 -0.045  0.135
## SRA        -0.361 -0.032  0.024  0.040 -0.002 -0.032
## RelStatus2 -0.051 -0.034 -0.076 -0.038  0.040  0.101  0.083
```

Summary Table Sex satisfaction

```r
class(m_Sex_Satis_1) <- "lmerMod"
class(m_Sex_Satis_2) <- "lmerMod"
class(m_Sex_Satis_3) <- "lmerMod"
stargazer(m_Sex_Satis_1, m_Sex_Satis_2, m_Sex_Satis_3, type = "text", digits = 2,
          star.cutoffs = c(0.05, 0.01, 0.001),report=('vc*stp'))
```

```
##
## ===================================================
##                           Dependent variable:
##                     ------------------------------
##                                Sex_Satis
##                        (1)        (2)       (3)
## ---------------------------------------------------
```

```
## HPP_9                  -0.81     -3.80*     -3.87*
##                        (1.46)    (1.83)     (1.76)
##                      t = -0.55  t = -2.07 t = -2.20
##                       p = 0.59  p = 0.04   p = 0.03
##
## GINI                              0.27*      0.31**
##                                  (0.11)     (0.11)
##                                 t = 2.37   t = 2.77
##                                 p = 0.02   p = 0.01
##
## GDP                              -0.06      -0.03
##                                  (0.10)     (0.09)
##                                t = -0.60 t = -0.37
##                                 p = 0.56   p = 0.72
##
## Sex2                                       5.53***
##                                             (1.40)
##                                           t = 3.96
##                                          p = 0.0001
##
## Age                                         -0.08
##                                             (0.06)
##                                          t = -1.39
##                                           p = 0.17
##
## SRA                                          1.27*
##                                             (0.52)
##                                           t = 2.47
##                                           p = 0.02
##
## RelStatus2                                  -0.27
##                                             (1.45)
##                                          t = -0.19
##                                           p = 0.86
##
## Constant              71.17***  61.90***   52.28***
##                        (1.15)    (6.35)     (7.01)
##                      t = 62.09  t = 9.74   t = 7.46
##                       p = 0.00  p = 0.00   p = 0.00
##
## ---------------------------------------------------
## Observations            1,857     1,857     1,840
## Log Likelihood      -8,702.76  -8,702.25 -8,611.41
## Akaike Inf. Crit.   17,413.52  17,416.50 17,242.81
## Bayesian Inf. Crit. 17,435.63  17,449.66 17,297.99
## ===================================================
## Note:                 *p<0.05; **p<0.01; ***p<0.001
```

Sex satisfaction by sex

```r
t.test(Sex_Satis ~ Sex, db, conf.int=TRUE)
```

```
##
##  Welch Two Sample t-test
```

```
##
## data:  Sex_Satis by Sex
## t = -3.7356, df = 780.54, p-value = 0.0002009
## alternative hypothesis: true difference in means is not equal to 0
## 95 percent confidence interval:
##  -8.361496 -2.600944
## sample estimates:
## mean in group 1 mean in group 2
##        67.68826        73.16948
```

```
group.CI(Sex_Satis ~ Sex, data = db, ci = 0.95)
```

```
##   Sex Sex_Satis.upper Sex_Satis.mean Sex_Satis.lower
## 1   1        70.24096       67.68826        65.13556
## 2   2        74.50707       73.16948        71.83189
```

```
cohen.d(Sex_Satis ~ Sex, data = db)
```

```
##
## Cohen's d
##
## d estimate: -0.2091386 (small)
## 95 percent confidence interval:
##      lower      upper
## -0.3123556 -0.1059216
```

Principal Component Analysis

PCA on the determinants of a good kiss

```
# Dataset with columns of interest
kiss_comp <- db_sample[,35:41]
kiss_comp <- cbind(kiss_comp,db_sample$Sex)
kiss_comp <- cbind(kiss_comp,db_sample$Country_Born)
kiss_comp <- cbind(kiss_comp,db_sample$HPP_9)
kiss_comp <- cbind(kiss_comp,db_sample$GINI)
kiss_comp <- cbind(kiss_comp,db_sample$GDP)
kiss_comp <- cbind(kiss_comp,db_sample$ID)
colnames(kiss_comp) <- c("GK_Breath","GK_Scent","GK_Taste","GK_Wet","GK_Contact",
                         "GK_Arous","GK_SynchStyle","Sex","Country","HPP_9","GINI",
                         "GDP","ID")

# Exclusion of missing data on the 7 variables of interest
for (i in 1:7) {
 kiss_comp <- kiss_comp[-which(is.na(kiss_comp[,i])),]
}

# Predictors
Sex <- as.factor(as.character(kiss_comp$Sex))
Country <- as.factor(as.character(kiss_comp$Country))
HPP_9 <- as.numeric(as.character(kiss_comp$HPP_9))
GINI <- as.numeric(as.character(kiss_comp$GINI))
GDP <- as.numeric(as.character(kiss_comp$GDP))

# Kaiser-Meyer-Olkin technique
```

```r
KMO(kiss_comp[,1:7])
```

```
## Kaiser-Meyer-Olkin factor adequacy
## Call: KMO(r = kiss_comp[, 1:7])
## Overall MSA =  0.75
## MSA for each item =
##     GK_Breath      GK_Scent      GK_Taste        GK_Wet    GK_Contact
##          0.74          0.71          0.79          0.80          0.72
##      GK_Arous GK_SynchStyle
##          0.71          0.82
```

```r
# Bartlett's Test of Sphericity
bart_spher(kiss_comp[,1:7])
```

```
##  Bartlett's Test of Sphericity
##
## Call: bart_spher(x = kiss_comp[, 1:7])
##
##      X2 = 3204.529
##      df = 21
## p-value < 2.22e-16
```

```r
# PCA
ncomp <- 2
kiss_pca <- prcomp(kiss_comp[,1:7], center=T, scale=T)
# Results
print(kiss_pca)
```

```
## Standard deviations (1, .., p=7):
## [1] 1.6496730 1.1200056 0.9071587 0.8533115 0.7610522 0.6920394 0.6441816
##
## Rotation (n x k) = (7 x 7):
##                     PC1        PC2         PC3        PC4         PC5
## GK_Breath     0.3706439 -0.4849616  0.03070620 -0.1499499  0.57552472
## GK_Scent      0.4176871 -0.4440279  0.11305754 -0.1193451 -0.01760598
## GK_Taste      0.4260376 -0.2841717 -0.02296307  0.2049687 -0.72037896
## GK_Wet        0.3524492  0.2278360 -0.47127072  0.7048798  0.21503909
## GK_Contact    0.3801467  0.3953525  0.45766694  0.1008459  0.26492302
## GK_Arous      0.3717342  0.4399418  0.38748245 -0.2042170 -0.17979989
## GK_SynchStyle 0.3158073  0.2917812 -0.63564931 -0.6103520 -0.02759481
##                        PC6         PC7
## GK_Breath      0.373608818  0.36495928
## GK_Scent      -0.340393859 -0.69654397
## GK_Taste      -0.007702767  0.41975436
## GK_Wet         0.133754499 -0.20195697
## GK_Contact    -0.564109067  0.30191502
## GK_Arous       0.615341509 -0.25582117
## GK_SynchStyle -0.172437410  0.08974262
```

```r
summary(kiss_pca)
```

```
## Importance of components:
##                           PC1    PC2    PC3    PC4     PC5     PC6     PC7
## Standard deviation     1.6497 1.1200 0.9072 0.8533 0.76105 0.69204 0.64418
## Proportion of Variance 0.3888 0.1792 0.1176 0.1040 0.08274 0.06842 0.05928
## Cumulative Proportion  0.3888 0.5680 0.6855 0.7896 0.87230 0.94072 1.00000
```

```r
# Barplot of the explained variances
fviz_screeplot(kiss_pca, addlabels = TRUE)
```

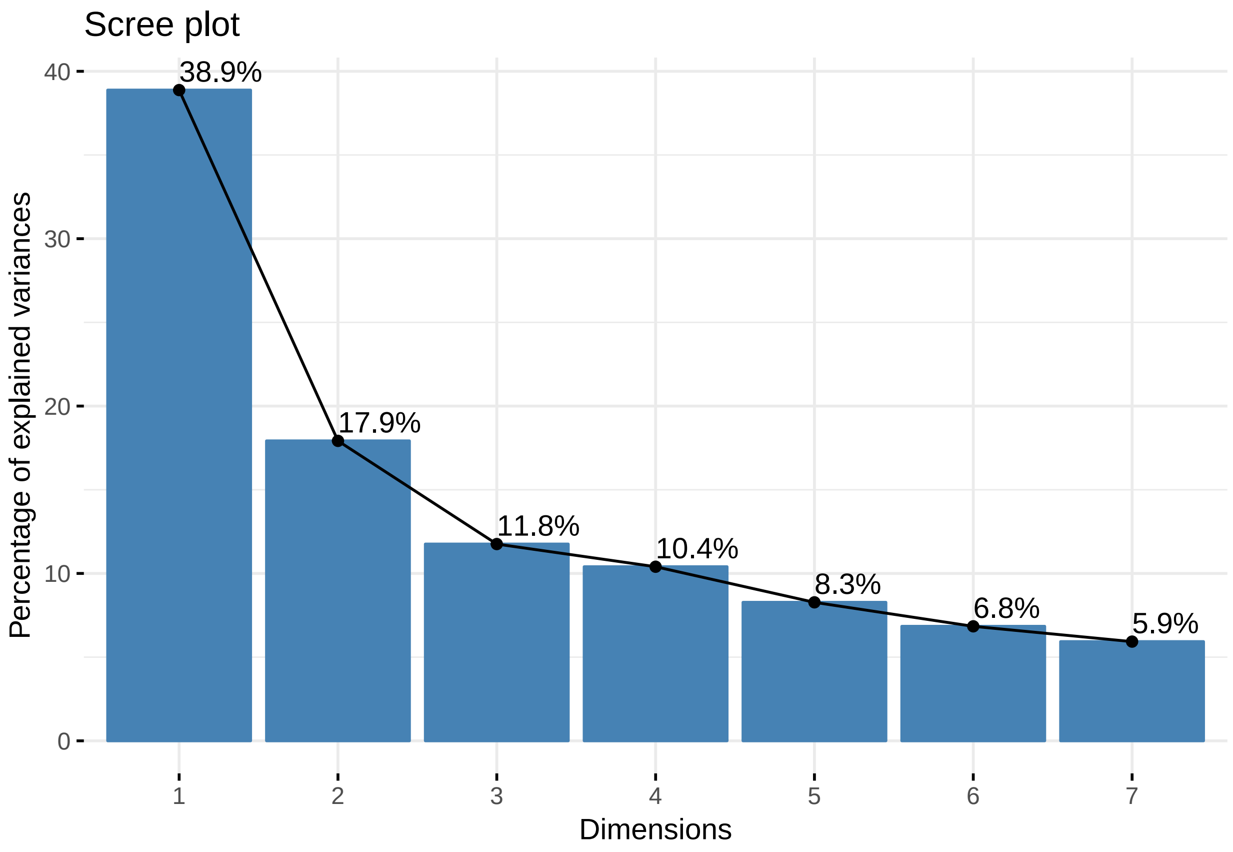


```r
# Graph of variables - colors using their contributions
fviz_pca_var(kiss_pca, col.var="contrib",
             gradient.cols = c("#00AFBB", "#E7B800", "#FC4E07"),
             repel = TRUE # Avoid text overlapping
)
```

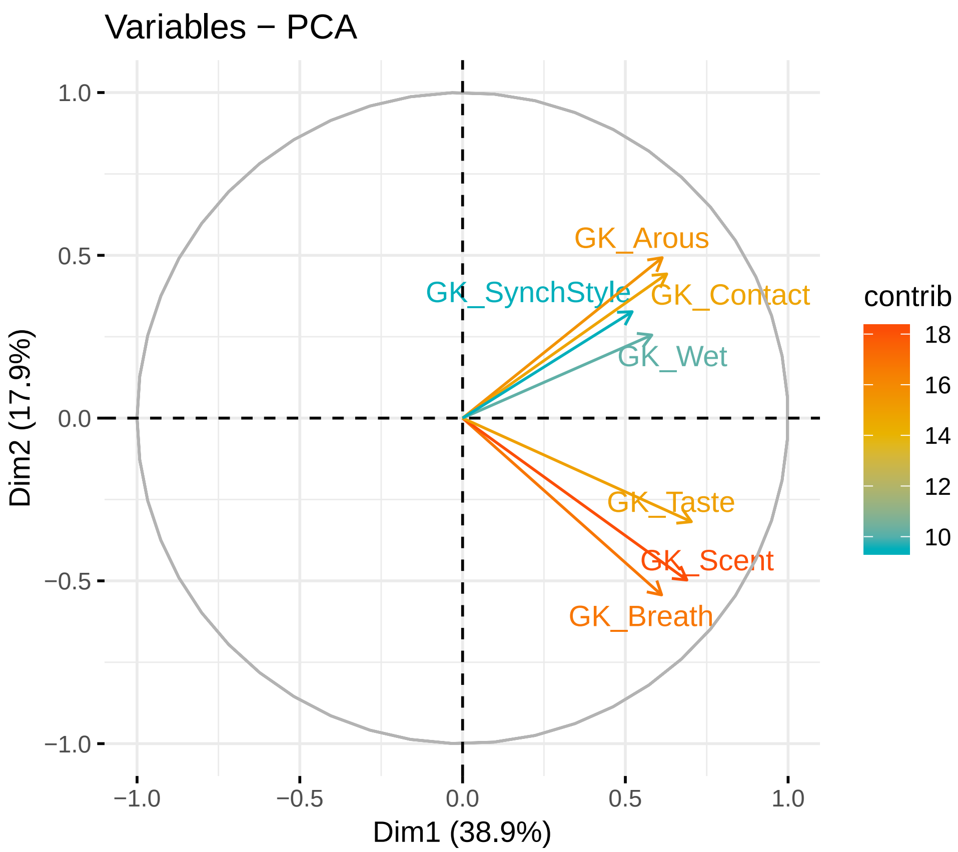


```r
# By sex
fviz_pca_ind(kiss_pca,
             geom.ind = "point",
             col.ind = Sex, # colors by groups
             palette = c("#00AFBB", "#E7B800"),
             addEllipses = TRUE, # Concentration
             legend.title = "Sex"
             )
```

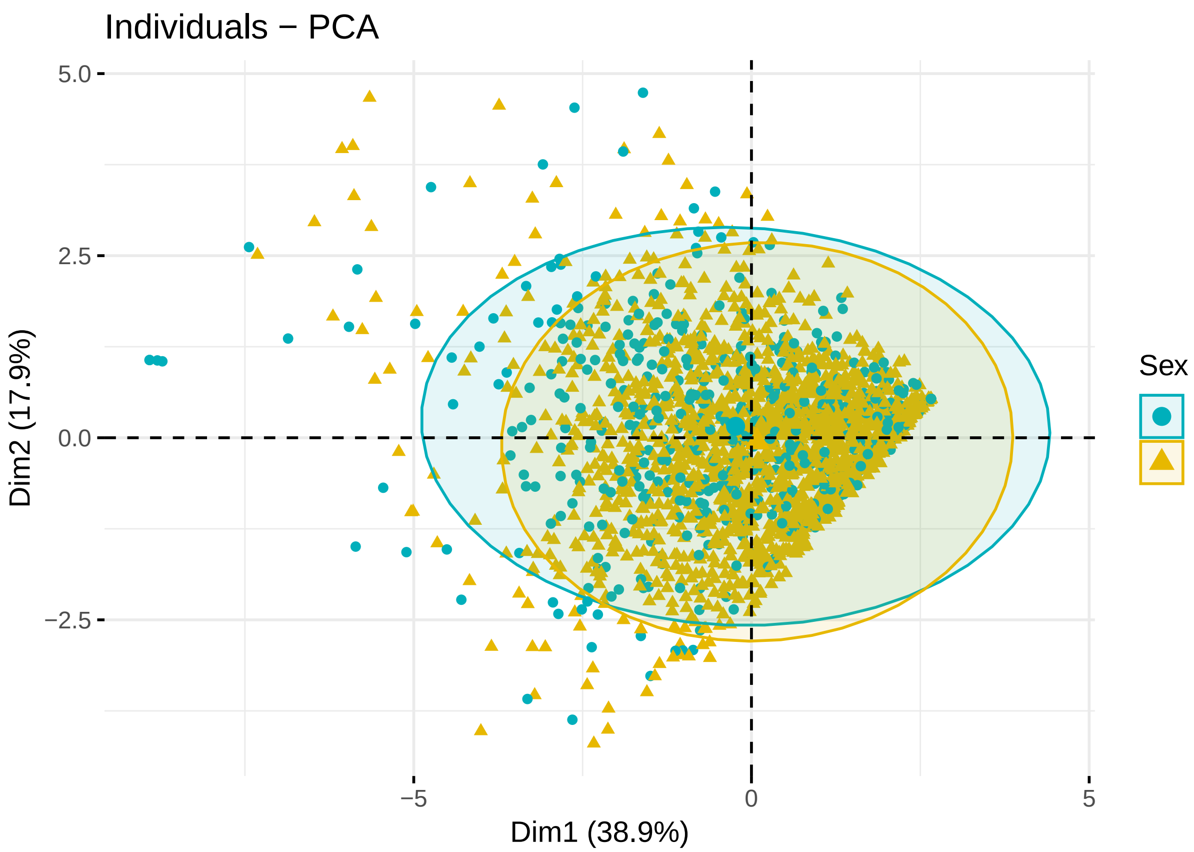

Varimax rotation

```
# Individuals' scores (using varimax rotation)
kiss_pca_rotated <- principal(kiss_comp[,1:7], nfactors=ncomp, rotate="varimax",
                              normalize=F, eps=1e-14)
kiss_pca_rotated
```

```
## Principal Components Analysis
## Call: principal(r = kiss_comp[, 1:7], nfactors = ncomp, rotate = "varimax",
##     normalize = F, eps = 1e-14)
## Standardized loadings (pattern matrix) based upon correlation matrix
##                 RC2  RC1   h2   u2 com
## GK_Breath      0.82 0.04 0.67 0.33 1.0
## GK_Scent       0.84 0.12 0.72 0.28 1.0
## GK_Taste       0.73 0.26 0.60 0.40 1.3
## GK_Wet         0.24 0.59 0.40 0.60 1.3
## GK_Contact     0.14 0.75 0.59 0.41 1.1
## GK_Arous       0.10 0.78 0.62 0.38 1.0
## GK_SynchStyle 0.15 0.60 0.38 0.62 1.1
##
##                        RC2  RC1
## SS loadings           2.01 1.97
## Proportion Var        0.29 0.28
## Cumulative Var        0.29 0.57
## Proportion Explained  0.51 0.49
## Cumulative Proportion 0.51 1.00
```

```
##
## Mean item complexity =  1.1
## Test of the hypothesis that 2 components are sufficient.
##
## The root mean square of the residuals (RMSR) is  0.11
##  with the empirical chi square  1192.23  with prob <  4.6e-252
##
## Fit based upon off diagonal values = 0.86
```

```
GK_rotated_PCs <- kiss_pca_rotated$scores
GK_rotated_PCs[1:5,]
```

```
##            RC2       RC1
## 1  0.003305789 0.2352164
## 2  0.910862993 0.3562293
## 3 -0.186638851 1.0597504
## 4 -0.559737772 0.7905664
## 5  0.688094111 0.8850645
```

```
# Table with the first 2 dimensions for each individual
GK_rotated_PCs <- cbind(kiss_comp$ID,GK_rotated_PCs[,1:2])
colnames(GK_rotated_PCs) <- c("ID","GK_rotated_dim2","GK_rotated_dim1")
# Merging with db_sample
db_sample_2 <- merge(db_sample, GK_rotated_PCs, by = "ID",all.x = TRUE)
```

Excluding participants from the new dataset

```
db_2 <- subset(db_sample_2, db_sample_2$Ex_Cross==0)
db_2$Country_Born <- droplevels(db_2$Country_Born)
db_2$Country_Res <- droplevels(db_2$Country_Res)
```

Models with variables from the PCA

Models dim 1 ("technique") of a good kiss

```
m_GK_rotated_dim1_1 <- lmer(GK_rotated_dim1 ~ HPP_9 +  (1|Country_Born), data=db_2)
summary(m_GK_rotated_dim1_1)
```

```
## Linear mixed model fit by REML. t-tests use Satterthwaite's method [
## lmerModLmerTest]
## Formula: GK_rotated_dim1 ~ HPP_9 + (1 | Country_Born)
##    Data: db_2
##
## REML criterion at convergence: 5047.4
##
## Scaled residuals:
##     Min      1Q  Median      3Q     Max
## -4.4363 -0.5868  0.1442  0.7154  2.2663
##
## Random effects:
##  Groups       Name        Variance Std.Dev.
##  Country_Born (Intercept) 0.008803 0.09382
##  Residual                 0.965808 0.98276
## Number of obs: 1795, groups:  Country_Born, 13
```

```
##
## Fixed effects:
##             Estimate Std. Error      df t value Pr(>|t|)
## (Intercept)  0.08387    0.03948 8.73710   2.125  0.06347 .
## HPP_9        0.21959    0.05006 7.80669   4.387  0.00247 **
## ---
## Signif. codes:  0 '***' 0.001 '**' 0.01 '*' 0.05 '.' 0.1 ' ' 1
##
## Correlation of Fixed Effects:
##       (Intr)
## HPP_9 0.255
```

```
m_GK_rotated_dim1_2 <- lmer(GK_rotated_dim1 ~ HPP_9 + GINI + GDP +  (1|Country_Born),
                            data=db_2)
summary(m_GK_rotated_dim1_2)
```

```
## Linear mixed model fit by REML. t-tests use Satterthwaite's method [
## lmerModLmerTest]
## Formula: GK_rotated_dim1 ~ HPP_9 + GINI + GDP + (1 | Country_Born)
##    Data: db_2
##
## REML criterion at convergence: 5064.4
##
## Scaled residuals:
##     Min      1Q  Median      3Q     Max
## -4.4419 -0.5935  0.1436  0.7121  2.2641
##
## Random effects:
##  Groups       Name        Variance Std.Dev.
##  Country_Born (Intercept) 0.01095  0.1046
##  Residual                 0.96583  0.9828
## Number of obs: 1795, groups:  Country_Born, 13
##
## Fixed effects:
##               Estimate Std. Error         df t value Pr(>|t|)
## (Intercept)  0.2083784  0.2868148 13.9062093   0.727    0.480
## HPP_9        0.1546792  0.0902675  7.7689085   1.714    0.126
## GINI        -0.0003055  0.0053119 10.7831332  -0.058    0.955
## GDP         -0.0039745  0.0044655 11.7384471  -0.890    0.391
##
## Correlation of Fixed Effects:
##       (Intr) HPP_9  GINI
## HPP_9 -0.153
## GINI  -0.900 -0.167
## GDP   -0.703  0.680  0.353
```

```
m_GK_rotated_dim1_3 <- lmer(GK_rotated_dim1 ~ HPP_9 + GINI + GDP + Sex + Age + RelStatus +
                              (1|Country_Born), data=db_2)
summary(m_GK_rotated_dim1_3)
```

```
## Linear mixed model fit by REML. t-tests use Satterthwaite's method [
## lmerModLmerTest]
## Formula: GK_rotated_dim1 ~ HPP_9 + GINI + GDP + Sex + Age + RelStatus +
##     (1 | Country_Born)
##    Data: db_2
```

```
##
## REML criterion at convergence: 5016.4
##
## Scaled residuals:
##     Min      1Q  Median      3Q     Max
## -4.4514 -0.5915  0.1382  0.7237  2.3146
##
## Random effects:
##  Groups       Name        Variance Std.Dev.
##  Country_Born (Intercept) 0.01025  0.1012
##  Residual                 0.95441  0.9769
## Number of obs: 1780, groups:  Country_Born, 13
##
## Fixed effects:
##               Estimate Std. Error         df t value Pr(>|t|)
## (Intercept)  1.198e-01  2.927e-01  1.514e+01   0.409  0.68799
## HPP_9        1.624e-01  8.885e-02  7.206e+00   1.827  0.10917
## GINI        -5.477e-04  5.235e-03  1.014e+01  -0.105  0.91872
## GDP         -4.556e-03  4.409e-03  1.109e+01  -1.033  0.32338
## Sex2        -3.206e-02  5.326e-02  1.773e+03  -0.602  0.54723
## Age          5.765e-03  2.133e-03  1.485e+03   2.702  0.00697 **
## RelStatus2  -1.038e-01  5.425e-02  1.771e+03  -1.913  0.05590 .
## ---
## Signif. codes:  0 '***' 0.001 '**' 0.01 '*' 0.05 '.' 0.1 ' ' 1
##
## Correlation of Fixed Effects:
##            (Intr) HPP_9  GINI   GDP    Sex2   Age
## HPP_9      -0.156
## GINI       -0.866 -0.163
## GDP        -0.672  0.681  0.358
## Sex2       -0.180  0.023  0.022  0.003
## Age        -0.207  0.012 -0.025 -0.041  0.128
## RelStatus2 -0.022 -0.024 -0.059 -0.030  0.016  0.099
```

Summary Table dim 1 ("technique") of a good kiss

```
class(m_GK_rotated_dim1_1) <- "lmerMod"
class(m_GK_rotated_dim1_2) <- "lmerMod"
class(m_GK_rotated_dim1_3) <- "lmerMod"
stargazer(m_GK_rotated_dim1_1, m_GK_rotated_dim1_2, m_GK_rotated_dim1_3, type = "text",
          digits = 2, star.cutoffs = c(0.05, 0.01, 0.001),report=('vc*stp'))
```

```
##
## ===================================================
##                          Dependent variable:
##                     -------------------------------
##                            GK_rotated_dim1
##                         (1)        (2)       (3)
## ---------------------------------------------------
## HPP_9                 0.22***     0.15      0.16
##                       (0.05)     (0.09)    (0.09)
##                      t = 4.39   t = 1.71  t = 1.83
##                     p = 0.0001  p = 0.09  p = 0.07
##
```

```
## GINI                             -0.0003   -0.001
##                                   (0.01)    (0.01)
##                                 t = -0.06 t = -0.10
##                                 p = 0.96  p = 0.92
##
## GDP                              -0.004    -0.005
##                                  (0.004)   (0.004)
##                                 t = -0.89 t = -1.03
##                                 p = 0.38  p = 0.31
##
## Sex2                                        -0.03
##                                            (0.05)
##                                           t = -0.60
##                                           p = 0.55
##
## Age                                        0.01**
##                                            (0.002)
##                                           t = 2.70
##                                           p = 0.01
##
## RelStatus2                                  -0.10
##                                            (0.05)
##                                           t = -1.91
##                                           p = 0.06
##
## Constant                0.08*      0.21      0.12
##                        (0.04)     (0.29)    (0.29)
##                       t = 2.12   t = 0.73  t = 0.41
##                       p = 0.04   p = 0.47  p = 0.69
##
## ---------------------------------------------------
## Observations            1,795      1,795     1,780
## Log Likelihood       -2,523.71  -2,532.18 -2,508.18
## Akaike Inf. Crit.     5,055.41   5,076.36  5,034.37
## Bayesian Inf. Crit.   5,077.38   5,109.31  5,083.73
## ===================================================
## Note:                  *p<0.05; **p<0.01; ***p<0.001
```

Models dim 2 ("sensory") of a good kiss

```r
m_GK_rotated_dim2_1 <- lmer(GK_rotated_dim2 ~ HPP_9 +  (1|Country_Born), data=db_2)
summary(m_GK_rotated_dim2_1)
```

```
## Linear mixed model fit by REML. t-tests use Satterthwaite's method [
## lmerModLmerTest]
## Formula: GK_rotated_dim2 ~ HPP_9 + (1 | Country_Born)
##    Data: db_2
##
## REML criterion at convergence: 5030.1
##
## Scaled residuals:
##     Min      1Q  Median      3Q     Max
## -5.4442 -0.4825  0.2291  0.7160  1.6950
##
```

```
## Random effects:
##  Groups       Name        Variance Std.Dev.
##  Country_Born (Intercept) 0.02586  0.1608
##  Residual                 0.95284  0.9761
## Number of obs: 1795, groups:  Country_Born, 13
##
## Fixed effects:
##             Estimate Std. Error       df t value Pr(>|t|)
## (Intercept)  0.02660    0.05479 11.70413   0.485    0.636
## HPP_9        0.04135    0.07018 11.38805   0.589    0.567
##
## Correlation of Fixed Effects:
##       (Intr)
## HPP_9 0.239
```

```
m_GK_rotated_dim2_2 <- lmer(GK_rotated_dim2 ~ HPP_9 + GINI + GDP +  (1|Country_Born),
                           data=db_2)
summary(m_GK_rotated_dim2_2)
```

```
## Linear mixed model fit by REML. t-tests use Satterthwaite's method [
## lmerModLmerTest]
## Formula: GK_rotated_dim2 ~ HPP_9 + GINI + GDP + (1 | Country_Born)
##    Data: db_2
##
## REML criterion at convergence: 5040.9
##
## Scaled residuals:
##     Min      1Q  Median      3Q     Max
## -5.4902 -0.4725  0.2276  0.7066  1.7150
##
## Random effects:
##  Groups       Name        Variance Std.Dev.
##  Country_Born (Intercept) 0.01037  0.1018
##  Residual                 0.95336  0.9764
## Number of obs: 1795, groups:  Country_Born, 13
##
## Fixed effects:
##              Estimate Std. Error        df t value Pr(>|t|)
## (Intercept) -0.431129   0.282599 13.719013  -1.526   0.1498
## HPP_9       -0.101519   0.088687  7.554403  -1.145   0.2873
## GINI         0.013536   0.005228 10.584750   2.589   0.0259 *
## GDP         -0.002747   0.004397 11.557759  -0.625   0.5442
## ---
## Signif. codes:  0 '***' 0.001 '**' 0.01 '*' 0.05 '.' 0.1 ' ' 1
##
## Correlation of Fixed Effects:
##       (Intr) HPP_9  GINI
## HPP_9 -0.153
## GINI  -0.900 -0.167
## GDP   -0.704  0.680  0.356
```

```
m_GK_rotated_dim2_3 <- lmer(GK_rotated_dim2 ~ HPP_9 + GINI + GDP + Sex + Age + RelStatus +
                              (1|Country_Born), data=db_2)
summary(m_GK_rotated_dim2_3)
```

```
## Linear mixed model fit by REML. t-tests use Satterthwaite's method [
## lmerModLmerTest]
## Formula: GK_rotated_dim2 ~ HPP_9 + GINI + GDP + Sex + Age + RelStatus +
##     (1 | Country_Born)
##    Data: db_2
##
## REML criterion at convergence: 4925
##
## Scaled residuals:
##     Min      1Q  Median      3Q     Max
## -5.7077 -0.4788  0.2089  0.6899  2.0097
##
## Random effects:
##  Groups       Name        Variance Std.Dev.
##  Country_Born (Intercept) 0.01905  0.1380
##  Residual                 0.90469  0.9512
## Number of obs: 1780, groups:  Country_Born, 13
##
## Fixed effects:
##               Estimate Std. Error         df t value Pr(>|t|)
## (Intercept) -1.074e+00  3.309e-01  1.480e+01  -3.245  0.00552 **
## HPP_9       -6.939e-02  1.059e-01  9.093e+00  -0.655  0.52853
## GINI         1.337e-02  6.084e-03  1.118e+01   2.197  0.04996 *
## GDP         -3.378e-03  5.093e-03  1.174e+01  -0.663  0.52006
## Sex2         2.929e-01  5.190e-02  1.773e+03   5.643 1.94e-08 ***
## Age          1.551e-02  2.088e-03  1.712e+03   7.430 1.70e-13 ***
## RelStatus2  -4.734e-02  5.285e-02  1.770e+03  -0.896  0.37048
## ---
## Signif. codes:  0 '***' 0.001 '**' 0.01 '*' 0.05 '.' 0.1 ' ' 1
##
## Correlation of Fixed Effects:
##            (Intr) HPP_9  GINI   GDP    Sex2   Age
## HPP_9      -0.157
## GINI       -0.867 -0.173
## GDP        -0.664  0.691  0.318
## Sex2       -0.156  0.016  0.020  0.002
## Age        -0.176  0.003 -0.024 -0.036  0.128
## RelStatus2 -0.020 -0.019 -0.048 -0.025  0.016  0.096
```

Summary Table dim 2 ("sensory") of a good kiss

```
class(m_GK_rotated_dim2_1) <- "lmerMod"
class(m_GK_rotated_dim2_2) <- "lmerMod"
class(m_GK_rotated_dim2_3) <- "lmerMod"
stargazer(m_GK_rotated_dim2_1, m_GK_rotated_dim2_2, m_GK_rotated_dim2_3, type = "text",
          digits = 2, star.cutoffs = c(0.05, 0.01, 0.001),report=('vc*stp'))
```

```
##
## ===================================================
##                           Dependent variable:
##                     -------------------------------
##                             GK_rotated_dim2
##                        (1)        (2)       (3)
## ---------------------------------------------------
```

```
## HPP_9                    0.04      -0.10     -0.07
##                         (0.07)    (0.09)     (0.11)
##                       t = 0.59  t = -1.14 t = -0.66
##                       p = 0.56  p = 0.26   p = 0.52
##
## GINI                             0.01**     0.01*
##                                  (0.01)     (0.01)
##                                 t = 2.59   t = 2.20
##                                 p = 0.01   p = 0.03
##
## GDP                              -0.003     -0.003
##                                  (0.004)    (0.01)
##                                 t = -0.62 t = -0.66
##                                 p = 0.54   p = 0.51
##
## Sex2                                       0.29***
##                                             (0.05)
##                                            t = 5.64
##                                          p = 0.0000
##
## Age                                        0.02***
##                                            (0.002)
##                                            t = 7.43
##                                            p = 0.00
##
## RelStatus2                                  -0.05
##                                             (0.05)
##                                           t = -0.90
##                                            p = 0.38
##
## Constant                 0.03      -0.43    -1.07**
##                         (0.05)    (0.28)     (0.33)
##                       t = 0.49  t = -1.53 t = -3.24
##                       p = 0.63  p = 0.13  p = 0.002
##
## ---------------------------------------------------
## Observations            1,795      1,795     1,780
## Log Likelihood      -2,515.06  -2,520.45 -2,462.49
## Akaike Inf. Crit.    5,038.12  5,052.90   4,942.98
## Bayesian Inf. Crit.  5,060.09  5,085.85   4,992.33
## ===================================================
## Note:                   *p<0.05; **p<0.01; ***p<0.001
```

Participant sex and importance of the sensory component of a good kiss

```
t.test(GK_rotated_dim2 ~ Sex, db_2, conf.int=TRUE)
```

```
##
##  Welch Two Sample t-test
##
## data:  GK_rotated_dim2 by Sex
## t = -4.2761, df = 770.49, p-value = 2.141e-05
```

```
## alternative hypothesis: true difference in means is not equal to 0
## 95 percent confidence interval:
##  -0.3444708 -0.1277057
## sample estimates:
## mean in group 1 mean in group 2
##     -0.14946024      0.08662799
```

```r
group.CI(GK_rotated_dim2 ~ Sex, data = db_2, ci = 0.95)
```

```
##   Sex GK_rotated_dim2.upper GK_rotated_dim2.mean GK_rotated_dim2.lower
## 1   1           -0.05400404          -0.14946024           -0.24491644
## 2   2            0.13809800           0.08662799            0.03515799
```

```r
cohen.d(GK_rotated_dim2 ~ Sex, data = db_2)
```

```
##
## Cohen's d
##
## d estimate: -0.2403011 (small)
## 95 percent confidence interval:
##      lower      upper
## -0.3453948 -0.1352074
```
